# Size reductions and genomic changes within two generations in wild walleye populations: associated with harvest?

**DOI:** 10.1111/eva.12987

**Published:** 2020-05-25

**Authors:** Ella Bowles, Kia Marin, Stephanie Mogensen, Pamela MacLeod, Dylan J. Fraser

**Affiliations:** ^1^ Concordia University Montreal QC Canada; ^2^ Golder Associates Montréal QC Canada; ^3^ University of Calgary Calgary AB Canada; ^4^ Cree Nation of Mistissini Mistissini QC Canada

**Keywords:** body size, fisheries‐induced evolution, genomics, harvesting, Indigenous knowledge, *Sander vitreus*, size‐at‐age, walleye

## Abstract

The extent and rate of harvest‐induced genetic changes in natural populations may impact population productivity, recovery, and persistence. While there is substantial evidence for phenotypic changes in harvested fishes, knowledge of genetic change in the wild remains limited, as phenotypic and genetic data are seldom considered in tandem, and the number of generations needed for genetic changes to occur is not well understood. We quantified changes in size‐at‐age, sex‐specific changes in body size, and genomic metrics in three harvested walleye (*Sander vitreus*) populations and a fourth reference population with low harvest levels over a 15‐year period in Mistassini Lake, Quebec. We also collected Indigenous knowledge (IK) surrounding concerns about these populations over time. Using ~9,000 SNPs, genomic metrics included changes in population structure, neutral genomic diversity, effective population size, and signatures of selection. Indigenous knowledge revealed overall reductions in body size and number of fish caught. Smaller body size, a small reduction in size‐at‐age, nascent changes to population structure (population differentiation within one river and homogenization between two others), and signatures of selection between historical and contemporary samples reflected coupled phenotypic and genomic change in the three harvested populations in both sexes, while no change occurred in the reference population. Sex‐specific analyses revealed differences in both body size and genomic metrics but were inconclusive about whether one sex was disproportionately affected. Although alternative explanations cannot be ruled out, our collective results are consistent with the hypothesis that genetic changes associated with harvesting may arise within 1–2.5 generations in long‐lived wild fishes. This study thus demonstrates the need to investigate concerns about harvest‐induced evolution quickly once they have been raised.

## 1 INTRODUCTION

Harvesting of wild populations can affect growth, body size, maturation, and population productivity (Heino et al., 2013; Heino & Godø, 2002; Hutchings, 2005), but it can also reduce genetic diversity (primarily through reducing population size) and select for different genotypes that underlie phenotypic traits (the latter commonly referred to as fisheries‐induced evolution, FIE) (Allendorf, England, Luikart, Ritchie, & Ryman, 2008; Hutchings & Fraser, 2008). In addition, intense harvesting could reduce the density of one population allowing for an increase in migrants from neighboring populations, which may then interbreed with and change the population structure of the harvested population (Allendorf et al., 2008). Because harvest‐induced genetic changes can affect population productivity, recovery, and persistence, assessing how quickly, to what extent, and under what circumstances such changes arise has become an emerging component of contemporary fisheries management (Heino, Pauli, & Dieckmann, 2015; Jorgensen et al., 2007; Law & Grey, 1989; Rowell, 1993, 2007).

Many studies have shown rapid phenotypic change toward smaller body size and size‐at‐age in harvested fish populations, though whether such changes are plastic responses (Law, 2007), genetic changes or both is a source of ongoing debate (Heino et al., 2015; Jorgensen et al., 2007; Sharpe & Hendry, 2009). Much of the empirical evidence that fishing causes rapid, genetically based phenotypic change comes from laboratory‐based studies (e.g., within three generations (van Wijk et al., 2013), or two to five generations (Therkildsen et al., 2019; Uusi‐Heikkilä, Sävilammi, Leder, Arlinghaus, & Primmer, 2017)). However, laboratory environments can introduce unintended selection pressures (possibly body condition, growth, maturation (Uusi‐Heikkilä et al., 2017)) and may not adequately depict the actual extent or rate of harvest‐induced change that wild fishes experience (Fraser et al., 2019; Walker, & Yates, 2019). Results from the few studies that have integrated phenotypic and genetic evidence in the wild suggest that harvest‐induced genetic change may occur within as little as one generation (Chebib, Renaut, Bernatchez, & Rogers, 2016), to four to eight (Allen, Bowles, Morris, & Rogers, 2017), or longer (Hutchinson, van Oosterhout, Rogers, & Carvalho, 2003; Therkildsen et al., 2013), though these studies were based on relatively limited genetic data and/or did not consider results by sex. Indeed, how genetic change from fishing may differentially affect males and females is understudied in fishes, despite that in many species, the sexes exhibit divergent, genetically based life histories (Fraser et al., 2019), and that harvest may affect the sexes differently (Hutchings & Rowe, 2008; Philipp et al., 2015). Some empirical results from the wild, and simulation models, suggest that female traits are more susceptible to harvest‐induced changes (Hixon, Johnson, & Sogard, 2013; Wang & Höök, 2009) and that sex‐selective harvest can occur due to sexual dimorphism (Lauer, Doll, Allen, Breidert, & Palla, 2008; Myers et al., 2014). Overall, there remains much to learn in nature about how fishing may drive genetic changes in the life history (e.g., body size, size‐at‐age, and sex) and genetic characteristics (e.g., population structure, genetic diversity, and composition) of wild populations.

While Western scientific methods (WSMs) are most often used to inform fisheries management, inclusion of Indigenous knowledge (IK) has become an integral complement to scientific knowledge for wildlife management and community‐based conservation (Berkes et al., 2000; Fraser, Coon, Prince, Dion, & Bernatchez, 2006; Polfus et al., 2014, 2016). Indigenous knowledge is defined as the “cumulative body of knowledge, practice and belief, evolving by adaptive processes and handed down through generations by cultural transmission, about the relationship of living beings (including humans) with one another and with their environment” (*sensu* Berkes et al., 2000). Importantly, IK provides extensive location‐specific knowledge, can detect changes in wildlife more quickly than WSM (Huntington, 2011), and often provides increased knowledge of environmental linkages (Chapman, 2007; Drew, 2005).

**FIGURE 1:**
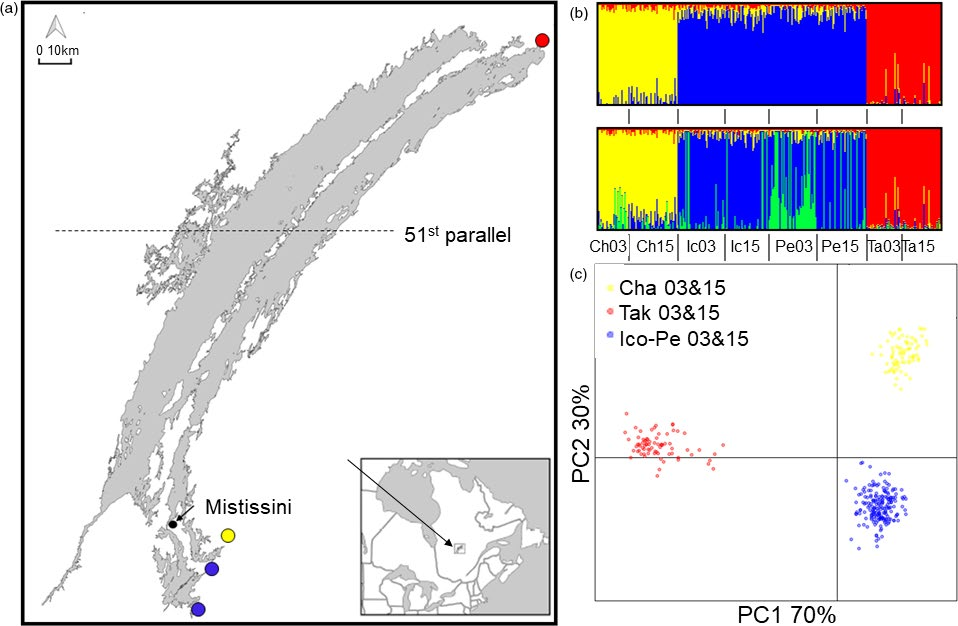
(a) Map of sampling sites: red is Takwa River, blue is Icon and Perch Rivers, green is a historical genotype in Perch River, yellow is Chalifour River. (b) ADMIXTURE results showing *K* = 3 (top) and *K* = 4 (bottom). (c) DAPC showing *k* = 3 (i.e., historical and contemporary sampling years are not separated statistically by DAPC)

Walleye (*Sander vitreus*) are important for commercial, sport, and Indigenous subsistence fisheries across North America (Bozek, Baccante, & Lester, 2011, 2015; Hansen, Carpenter, Gaeta, Hennessy, & Vander Zanden, 2015; Scott & Crossman, 1979). Mistassini Lake in northern Quebec, Canada, is the province's largest natural lake (161 km long, 2,335 km^2^, 183 m maximum depth), is in Grand Council of the Crees land, *Eeyou Istchee*, and is considered to be largely pristine (minimal mining, forestry, development; no known invasive species) (Fraser et al., 2006; Marin, Coon, & Fraser, 2017). The motivation behind this study was observations by Cree elders and fishers of reduced body size and catch rates in walleye populations in three of Mistassini Lake's southern tributaries that are close to the community, and a desire by the community to determine whether management actions were needed. This is particularly important, since walleye is a preferred subsistence food source and more easily harvested than other fish, especially close to the community. We also studied a fourth river at the northeastern tip of the lake, where the population was perceived to be largely unaffected by fishing until very recently (~2015, IK, see methods). Subsistence harvest takes place on the rivers during spawning in the spring, and walleye from different rivers comprise a mixed‐population fishery in the lake during the summer, both recreationally and for subsistence (Tables S1 and S2). However, recreational non‐Cree fishers are only permitted to fish below the 51st parallel when they are without a Cree guide (Figure 1), fishing by Cree is mostly in the south (See fishing pressure section below for details), and the genetically distinct populations that contribute most to the mixed summer fishery are those from the rivers of concern (i.e., southern populations stay close to their spawning rivers to feed (Dupont, Bourret, & Bernatchez, 2007)). Documented catch by non‐Cree fishers without a guide has not increased between 1997 and 2015 (Table S2), but we do not have data on direct or latent mortalities due to local fishing derbies. In addition, the human population and the number of households in Mistissini almost doubled between 1997 and 2016 (Table S2). Cumulatively, this information indicates an increase in fishing pressure in the southern rivers.

Using tissue samples and body size measurements collected in 2002/03 (Dupont et al., 2007) (“historical”) and between 2015 and 2017 (“contemporary”), we tested the general hypothesis that harvesting over a period of 1–2.5 generations (based on ages of spawners in the southern rivers of Mistassini Lake (supplementary data, Dupont et al., 2007)) was sufficient to generate coupled phenotypic and genetic changes in wild walleye populations. Specifically, we predicted that, in association with recent, increased fishing effort in Mistassini Lake, the following should be evident within the southern, harvested rivers but not in the northern river with limited harvesting, when comparing contemporary versus historic samples: (a) reduced body size (total length and mass); (b) reduced size‐at‐age; (c) changes to population structure such as collapsing/homogenization of between‐river population structure; (d) reductions in genetic diversity and effective population size; (e) signatures of selection, with putatively selected loci related to growth, body size, and/or maturation; and (f) greater reductions in body size, size‐at‐age, and stronger signatures of selection in females than in males, as a sexually dimorphic species with larger females than males. As one of the relatively few studies incorporating genomic and phenotypic data in wild populations to date, and the first to suggest rapid genetic change in a long‐lived species, this study could be used to inform population genomic parameters and monitoring practices for the sustainable harvest and management of other similar long‐lived species.

## 2 MATERIALS AND METHODS

### 2.1 Fishing pressure and Indigenous knowledge

Currently, there is no mechanism in place for Indigenous fishers to report the number of fish caught in Mistassini Lake. Thus, to establish trends in fishing pressure, fish abundance, and body size, we conducted semi‐directed interviews as in Fraser et al. (2006), during February and July 2018, with 17 elders and fisherman (30–79 years of age, with 13 respondents > 40 years) (see Table S1 for questions asked). Importantly, as per Tengö et al. (2017) elders and fishers were not chosen randomly. They were well‐respected authorities on matters of fishing within the community, and there were few other community members with similar knowledge. Answers were not used for questions where respondents explicitly stated a lack of knowledge, as per Gagnon and Berteaux (2009), and the frequency of respondents for a given answer has been provided, using the total number of respondents for that question as the denominator. If the majority of respondents gave similar answers for frequency‐based questions, we took this into consideration; because IK is rich in narrative, we also considered relevant pieces of information separately. In addition, we obtained census numbers for all people in the community close to the lake and the number of fish caught by non‐Cree fishers for a subset of years (Table S2). Informed consent was obtained prior to each interview and for each of the interviewees (ethics certificate no. 30,008,247).

Rivers included in the study were Chalifour, Icon, and Perch in the south and Takwa in the north. Communicated incidentally by two IK respondents and by several Cree fishers and community members in 2017 and 2018, Takwa was perceived to be relatively unaffected by fishing until ~ 2015 when larger boat motors made access easier.

### 2.2 Fish sampling

Fish were sampled during spawning (after ice‐off: mid‐May in the south and early June in the north) at spawning rivers in 2002 and 2003 by Dupont et al. (2007) (historical), and in 2015, 2016, and 2017 (contemporary) by us (see Table 1 for sample sizes). Sampling was collaborative with subsistence fishers for 2015–2017. Walleye were captured via angling using the same lures and a combination of boats and shore fishing, from the same locations within rivers, for both historical and contemporary sampling (Table 1). Catch per unit effort was not available for historic samples or collaborative sampling and is therefore not included here for contemporary sampling. After capture, fish were immediately placed in freshwater baths with aerators. From each walleye, we collected total and fork length (TL ± 1 mm), wet mass (± 50 g), sex (M, F, U (unknown, either spawned out or premature)), and a tissue sample for genetics; otoliths were collected from a random subsample. Live walleye were returned to the water near the location of capture. Opercular bones but not otoliths were collected for aging for historic samples (Table 1), and this was done before this study, for Dupont et al. (2007); 2015 and 2017 otoliths were aged at the Wisconsin Cooperative Fishery Unit, US Geological Service, University of Wisconsin, Stevens Point, USA. Otoliths were aged by two experienced readers; if they disagreed on an age, they examined the structure together to agree upon one for that structure. No walleye were aged using both opercular bones and otoliths.

**TABLE 1:** Details of sample sizes for walleye caught in each tributary of Mistassini Lake for each sex and year for each analysis, as well as whether samples were caught from a boat or on shore

River	Year	Boat or shore fishing	Body size (2002/03 samples grouped)b	Size‐at‐age	Genomic after removing aberrant individuals (only 2003 and 2015 samples used)^c^
Chalifour	2002	Primarily boat^a^	164(M), 14(F)	21(M), 11(F)	
	2003	Primarily boat^a^			22(M), 8(F)
	2015	Primarily boat	118(M), 14(F), 44(U)	18(M), 3(F)	39(M), 9(F), 1(U)
	2016	Primarily boat	132(M), 29(F), 2(U)		
	2017	Primarily boat	96(M), 12(F), 9(U)	10(M),4(F)	
Perch	2002	Not available	113(M), 43(F)	17(M), 37(F)	
	2003	Not available			24(M), 24(F)
	2015	Primarily boat	34(M), 13(F), 13(U)	3(M), 3(F)	31(M), 11(F)
	2016	Primarily shore	78(M), 26(F), 9(U)		
	2017	Primarily shore	12(M)		
Icon	2002	Not available	77(M), 43(F)	27(M), 34(F)	
	2003	Not available			23(M), 24(F)
	2015	Primarily shore	106(M), 8(F)	13(M), 3(F)	37(M), 7(F)
	2016	Primarily shore	156(M), 13(F), 1(U)		
	2017	Primarily shore	38(M), 1(F), 1(U)		
Takwa	2002	Primarily boat^a^	64(M), 81(F), 26(U)	28(M), 15(F)	
	2003	Primarily boat^a^			17(M), 16(F), 2(U)
	2015	Boat	116(M), 15(F), 19(U)	10(M), 11(F)	20(M), 19(F)
	2016	Boat			
	2017	Boat	51(M), 22(F), 76(U)		

^a^ Takwa and Chalifour spawning grounds are only accessible by boat; thus, while we do not have record of exactly how fishing was conducted, boat can be inferred.

^b^ Individuals with unknown sex were not used in body size models, and nor were categories with any fewer than 8 observations.

^c^ We extracted DNA from 371 walleye (historical *n* = 173 from 2003, contemporary *n* = 198 from 2015), and Icon and Perch were analyzed as a single population.

### 2.3 Body size at spawning and size‐at‐age

We modeled both body size (total length and mass) and size‐at‐age in this study because we had a far greater sample size for body size estimates than aged samples, and mass estimates had not been correlated with historic aged samples. Thus, evaluating body size allowed us to investigate changes in length and mass on a per‐river, per‐sex, and per‐year basis.

#### 2.3.1 Body size

We used multiple regressions and ANOVA in R (R Core Team, 2017) to test our prediction that body size of breeding adults had been reduced in southern rivers between 2002/03 and each 2015, 2016, and 2017. Year was set as a factor. Error was normally distributed for total length (TL), and mass was log‐transformed to improve fit of the error term. Our full model for each TL and mass (*Y_i_*) included the following.

$$Y_{i}=\beta_{0}+\beta_{1}{\text{Year}}_{i}+\beta_{2}{\text{River}}_{i}+\beta_{3}{\text{Sex}}_{i}+\beta_{4}{\text{Year}}_{i}\times {\text{River}}_{i}+\beta_{5}{\text{Year}}_{i}\times {\;\text{Sex}}_{i}+\beta_{6}{\text{River}}_{i}\times {\text{Sex}}_{i}+\beta_{7}{\text{Year}}_{i}\times {\text{River}}_{i}\times {\text{Sex}}_{i}+e_{i}$$

To determine the best model, we used backward stepwise model selection and AIC (Akaike, 2018, 1974). Significance was detected at an alpha of 0.05, and all multicomparison *p*‐values were adjusted using the false discovery rate (FDR) method for 64 planned contrasts (Tables S3 and S5) (Benjamini and Hochberg 1995).

There were insufficient samples collected across locations in 2002 and 2003 to use these years independently for body size analysis. Since no population genetic structure existed between 2002 and 2003 within rivers (Dupont et al., 2007), they were combined for all analyses, and denoted as 2002/03. In addition, in 2017 we were unable to collect any female walleye from Perch River, nor a sufficient number of female walleye in Icon River to be able to use them in length/mass models (see Table 1 for sample numbers).

#### 2.3.2 Size‐at‐age

To test our prediction of reduction in size‐at‐age in the southern populations relative to the northern one through time, we used a Bayesian hierarchical regression model. We used Bayesian as opposed to frequentist modeling to account for possible bias due to sampling gear, as well as small and variable sample sizes for aging structures across rivers. In addition, only total length was modeled because mass data were not included in the historical dataset containing age information. We assumed walleye total length (TL) was normally distributed such that:

$${\text{TL}}_{i}∼\text{normal}\left(\mu_{i},\sigma \right).$$

with shape parameters $\mu_{i}$ and σ representing the mean and standard deviation for walleye total length, respectively. Mean total length for the *i*th walleye was then modeled using linear regression:

$$\mu_{i}=\beta_{0}+\beta_{1}{\text{Age}}_{i}+\beta_{2}{\text{Location}}_{i}+\beta_{3}{\text{History}}_{i}+\beta_{4}{\text{Sex}}_{i}+\beta_{5}{\text{History}}_{i}\times {\text{Location}}_{i}$$

We used vague normal priors for all β coefficients and modeled hyperpriors for age and sex by river. Location (southern rivers versus northern river) and history (contemporary versus historic samples) were coded as categorical variables.

The Bayesian model was run using JAGS version 4.3.0 (Plummer, 2003) in R, using *rjags* and *run.jags* (Denwood, 2016). We described the posterior distribution for the model using four MCMC chains. Starting parameter values for each chain were jittered. Each chain took 20,000 samples of the posterior, thinned at a rate of 50. The adaption period was 1,000 iterations, and a burn‐in rate of 50% was used, for a total chain length of 2,050,00. We evaluated MCMC chain convergence by visual inspection of trace plots to assess mixing. Additionally, we ensured that each parameter had effective sample sizes > 1,000 and that they passed the Gelman–Rubin diagnostic test with potential scale reduction factors (PSRF) <1.1 suggesting convergence on a common posterior mode (Gelman et al., 2013).

### 2.4 Sequencing

DNA was extracted using a modified Qiagen blood and tissue kit protocol (Qiagen Inc., Valencia, CA) (see Table 1 for sample sizes) and was sequenced using individual‐based genotyping by sequencing (GBS). Libraries for Ion Proton GBS were prepared using the procedure described by Mascher, Wu, Amand, Stein, and Poland (2013) at IBIS, Université Laval, Québec, Canada, with modifications described in Abed, Légaré, and Pomerleau (2018, 1974). Libraries were prepared for sequencing using an Ion CHEF, Hi‐Q reagents, and P1 V3 chips (Thermo Fisher), and the sequencing was performed for 300 flows. Enzymes used to cleave the DNA were rare cutter *pst1* and frequent cutter *msp1*.

Single nucleotide polymorphisms (SNPs) were determined from raw sequence reads using the *stacks* pipeline v1.45 (Catchen, Hohenlohe, Bassham, Amores, & Cresko, 2013), and de novo sequence alignment, on the supercomputer Guillimin from McGill University, managed by Calcul Québec and Compute Canada. Preprocessing of fastq files was completed using fastQC (https://www.bioinformatics.babraham.ac.uk/projects/fastqc/) to assess read‐quality before and after using cutadapt (Martin, 2011) to trim any remaining adapters and remove sequences < 50bp. Our stacks parameter optimization method was similar to Mastretta‐Yanes, Arrigo, and Alvarez (2015), but we did not estimate error rate because we did not have enough positive controls to do so. Final stack parameters included default settings with the following custom options: within *process_radtags*, 80bp trim length; ustacks, SNP model, alpha = 0.1 for SNP calls, ‐m = 7; *cstacks*, ‐*n* = 3; *rxstacks*, log‐likelihood cutoff = −30 for SNP calls; *populations*, log‐likelihood cutoff of −30 for SNP calls, choose single SNP, maf = 0.01, ‐*r* = 0.8. We ran *populations* twice. First, we used the parameters listed here, and generated a blacklist of loci consisting of loci with *F*_IS_ < −0.3. We then reran *populations* with the same parameters listed here using this blacklist, with ‐p 6/8 (all years and rivers separately) and ‐p 4/6 (Icon–Perch merged); the numerator is the total number of populations required to have that locus, while the denominator is the total number of populations in the population map. All years and rivers were used initially to find the best‐supported population structure and to discover any changes that were occurring between rivers and years. Once the appropriate population structure was determined, Icon–Perch were merged (see Population structure section of Results below for a full explanation). No negative controls produced stacks, and all positive controls assigned to the correct populations using discriminant analysis of principal components (DAPC) in Adegenet (Jombart, 2008; Jombart, Devillard, & Balloux, 2010). After quality trimming and filtering, an average of 8,457 (for ‐p‐4/6) and 8,728 (for ‐p 6/8) high‐quality SNPs were used to estimate population structure, genetic diversity, and effective population size (Ne). See Table 2 for a summary of the number of loci, SNPs, and sequencing coverage at each filtering stage.

**TABLE 2:** Summary of Mistassini Lake walleye sequence statistics after each stage of filtering using *stacks*. Loci identified here are *stacks* loci

	After cutadapt, loaded into *process_radtags*, total sequences	After *process_radtags*, retained reads, number (percentage)	After *ustacks* filtering: average (min, max)	*sstacks_rx*	*populations_rx* (all rivers and years considered separately, no positive controls, samples of unknown origin removed)
Plate 1	364,678,780	322,853,622 (88.5%)	NA	NA	NA
Plate 2	374,785,960	334,978,264 (89.4%)	NA	NA	NA
Plate 3	347,655,212	297,199,476 (85.5%)	NA	NA	NA
Plate 4	408,731,443	360,381,408 (88.2%)	NA	NA	NA
Number of reads per individual (for 371 individuals)	NA	average 3,478,507, STDEV (1,624,881)	2,617,476 (175,372, 7,342,579)	NA	NA
Depth of coverage per individual	NA	NA	23.8 (7, 677)	NA	NA
Number of loci	NA	NA	NA	244,712	50,319
Number of SNPs	NA	NA	NA	NA	8,728
Mean depth of coverage for each SNP across all individuals	NA	NA	NA	NA	26 (STDEV 14, min 7, max 118)

Note

“*rx*” appended to the program module name indicates that the statistic presented was at the end of the analysis completed by that module, following log‐likelihood filtering and correction using *rx_stacks*

### 2.5 Population structure

To test our prediction that harvesting in the southern populations would change genetic population structure, potentially homogenizing structure between southern rivers over time, we assessed population structure using DAPC, ADMIXTURE (Alexander, Novembre, & Lange, 2009), and genetic distance (*F*_ST_) (using GenoDive and 999 permutations, Meirmans & Van Tienderen, 2004; Weir & Cockerham, 1984). The optimal number of principal components (PCs) to retain for DAPC was determined using the xval procedure, using n/3 (recommended by the manual) as the maximum number of PCs allowable, and 500 replicates. The population grouping that best fit the data was assessed using Bayesian information criterion (BIC) for DAPC, while for ADMIXTURE analysis we used cross‐validation (CV) and 500 bootstrap replications. Both analyses were completed at least four times using different bootstrap values and numbers of replicates to ensure results were stable. Based on DAPC (when all rivers and years were considered separately), it was clear that 28 individuals, primarily sampled in Icon and Chalifour Rivers, were from different unsampled genetic source populations. We removed these individuals, reran the *populations* module of stacks, and conducted all subsequent analyses using this reduced dataset (i.e., for analyses where we show results for years and rivers considered separately and also where Icon and Perch were merged).

### 2.6 Removing loci potentially under selection

Global outlier loci (loci putatively under selection) were detected using PCAdapt (Luu, Bazin, & Blum, 2017), using the scree plot method to determine the best number of PCs (K) to retain (Jackson, 1993), and Mahalanobis distance with alpha = 0.1 to determine outliers. PCAdapt does not use predefined population structure, but instead ascertains structure based on PCA. The program detects outliers based on how they relate to the structure of populations on the PCA (i.e., the distance between a point and a distribution). After removing the outlier loci from the dataset, the effect of linkage disequilibrium (LD) on population structure was assessed by finding markers that were in LD (*r* ^2^ = 0.7) using plink v1.9 (Chang et al., 2011, 2015). We removed these markers (*n* = 507) and reanalyzed population structure with DAPC. Since linked loci had no effect on structure, they were retained for all subsequent analyses. Genetic diversity, *F*_ST_, and Ne analyses were completed both including and excluding global outlier loci—while there was little difference between results including or excluding outlier loci for genetic diversity and Ne, the magnitude of *F*_ST_ was greater with outlier loci included, and the conclusion changed in one case for *F*_ST_. Thus, we have included only results for neutral loci here for these two metrics.

### 2.7 Genetic diversity (H_E_) and Ne

To test the prediction that genetic diversity (expected heterozygosity, H_E_) and Ne were reduced over time in southern populations, genetic diversity estimates were obtained using the *populations* model of *stacks*, and per‐generation Ne (5‐ to 7‐year generation time) was estimated using the linkage disequilibrium method in NeEstimator v2.01 (Do, Waples, & Peel, 2014), requiring a minimum minor allele frequency of 0.02. Estimates were per river, for each of 2003 and 2015, with males and females combined (see genomic samples in Table 1). Ne estimates and confidence intervals were corrected for linkage by correcting for chromosome number according to Waples, Larson, and Waples (2016).

### 2.8 Signatures of selection

To test the prediction that signatures of selection would be most evident between timepoints within southern and not northern river(s) and that putatively selected loci would be associated with relevant biological processes, analyses to determine outlier loci were conducted with PCAdapt using the method described above. We chose to use PCadapt instead of other programs for outlier detection for two reasons. First, only two “populations” were sampled, and the authors of OutFlank do not recommend its use in these situations (Whitlock & Lotterhos, 2015). Second, the biological scenario in our populations is divergence and admixture. In this scenario, all other outlier programs that could be used with our data (SNPs not haplotypes) have between 20% and 40% false discovery rates (PCAdapt has 10%) (Luu et al., 2017). Analyses were conducted for sexes both combined and separately: (a) for all populations combined, (b) for southern rivers only, and (c) within each population. For all analyses, except when all populations and years were included, the *populations* module of *stacks* was rerun including only the populations and/or sexes that were being contrasted, specifying that loci had to be present in both populations (see Table S8 for sample sizes and numbers of loci in each analysis). For sex‐based analysis of the full dataset including all rivers and years, loci were required to be present in 6 of 8 populations.

To determine possible functions for outlier loci, for all within‐river historic–contemporary contrasts for which there were outlier loci, FASTA files were blasted, mapped, and annotated using blast2go (Götz et al., 2008). Default parameters were used with the following custom choices: proprietary cloudblast, fast‐blast, UniProtKB/Swiss‐Prot (Swissprot_v5) database, blast e‐value 1.0E‐5, 10 blast hits, and filtered GO by taxonomy taking only matches to animals (Metazoa).

## 3 RESULTS

### 3.1 Fishing pressure and Indigenous knowledge

Of 17 elders and fishers, most reported reductions in the size and number of walleye caught in the lake (15 and 14 respondents, respectively, or 94% and 93%, respectively, after respondents who did not know were removed) within the last 5–20 years (Table 3). Eleven respondents expressed concerns directly about overfishing, fishing during spawning, or taking of too many fish during spawning. There was no consistent change in the number of fish caught by non‐Cree fishers between 1997 and 2015 (virtually the same in 1997, 2011, and 2015, but 54% higher in 2003) (Table S2), but the community of Mistissini (3,724 people in 2016) grew by ~ 29% between 1997 and 2011, and the population and number of households in the community increased by ~ 50% between 1997 and 2016 (Table S2). In addition, while there are currently no data on the number of fishers in the community nor the proportion of the population that fishes, the majority of fishers (16/17) fished in the southern area of concern in the lake, more than double than in all other areas of the lake except Takwa River (where 9 of 17 fishers fished) (Table 3).

**TABLE 3:** IK for 17 fishers with > 25 years of walleye fishing experience on Mistassini Lake

Observation	Trend	Timeline of observation	No.	Freq
Area fished	Region close to community	Current time	16	0.94
	Far northeast, close to northern reference river	Current time	9	0.53
	Other areas of the lake	Current time	≤7	0.41
Size of fish	Smaller	5–25 years^a^	12	0.71
	Smaller in the south	15 years	3	0.18
	No change in average, but Takwa River fish are smaller	5 years	1	0.06
	Different sizes in different seasons	within years	1	0.06
	Not sure		1	0.06
Abundance of walleye	Decreasing	5–25 years	13	0.76
	Fewer in the south but not in the north		1	0.06
	No change	n/a	1	0.06
	Does not know	n/a	2	0.12
Other concerns about health of walleye	Fishing during spawning or taking too many during spawning		6	
	Use of snares to fish in the spring		3	
	Night fishing, taking too many		2	
	Overfishing		5	
	Damage to fish after being handled		1	
	Fishing derbies causing many dead fish		2	
	Pike are after walleye eggs		2	
	Pollution in lake, dirty water		2	
	No other concerns, or they will be fine		8	

Note

No. is the number of respondents for that answer, and Freq is the frequency of respondents using the number of respondents for the observation as the denominator. Frequency is not given for the observation for which respondents could respond for multiple trends.

^a^ Two of the respondents indicated 20 years and 20–25 years, respectively. All other respondents indicated 15 years or less.

### 3.2 Body size at spawning and size‐at‐age

#### 3.2.1 Body size

**FIGURE 2:**
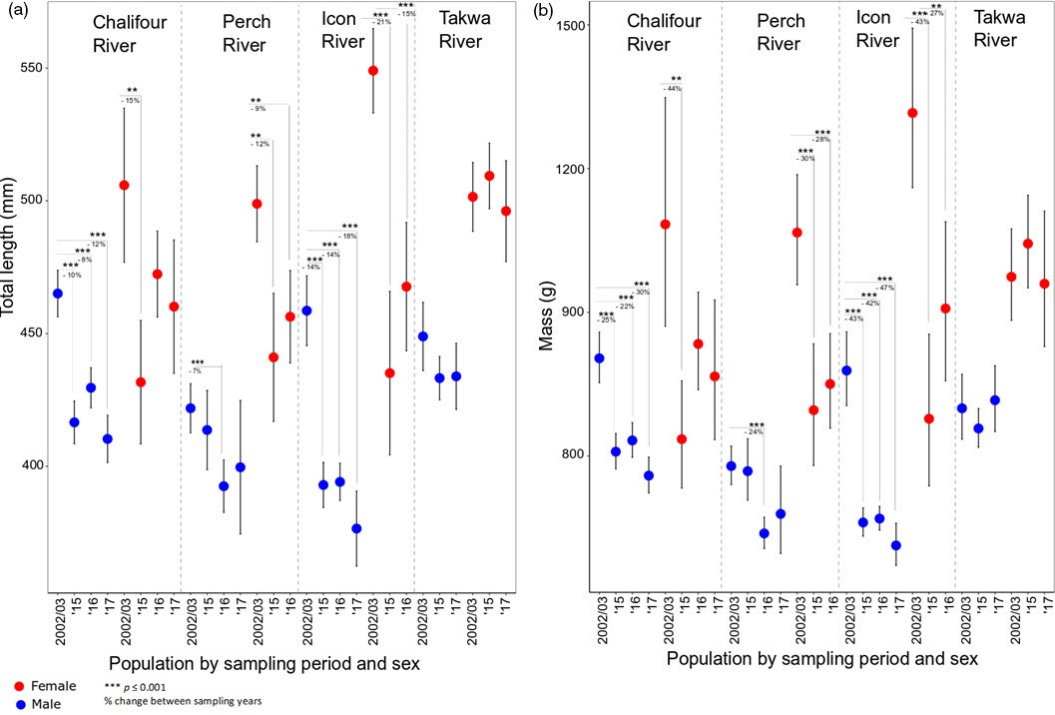
Least squares means (± 95% CI) of (a) total length and (b) mass for male and female walleye between 2002/2003, 2015, 2016, and 2017 in the four rivers surveyed. There was also a significant change between 2016 and 2017 (*p* = .0092 for TL and *p* = .003 for mass) for male fish in Chalifour, but this was not shown for clarity

Our prediction that body size would be reduced in southern populations within a 1‐ to 2.5‐generation period was supported. The main effects, year, river, and sex all had a significant effect on total length and mass (Table 4), and the best‐fit models for each TL and mass each contained a three‐way interaction between year, sex, and river. AIC was > 10 better for the full model for TL and > 6 better for mass. Both regression models were significant (TL, *R* ^2^ adj = 0.418, *F*_27, 1,486_ = 41.24, *p* < .001; mass *R* ^2^ adj = 0.3907, *F*_27, 1,481_ = 36.81). Mean TL and mass decreased significantly for both sexes between 2002/2003 and each of 2015, 2016, and 2017 in the southern rivers (TL 7%–21% and mass 22%–47% reductions), except for Perch River males in two contrasts and Chalifour females in two contrasts. While there was no significant change between 2002/2003 and each of 2016 and 2017 years for females in Chalifour, the trend in decline remained clear (Figure 2); lack of significance may relate to low female sample size in this river (Table 1). Indeed, the trend for Chalifour females was particularly evident when contrasted to Takwa (the reference northern river), where mean sizes of fish were consistent across all sampling years. Finally, a sex bias for more males than females being captured at spawning sites was consistent for all sampling years and for all rivers, including Takwa (Figure S1).

**TABLE 4:** Analysis of variance table for best fit (full) model for each walleye total length (TL) and mass, with response log(mass). Years are each 2002/2003, 2015, 2016, and 2017. Rivers are Chalifour, Icon, Perch, and Takwa

	*df*	Sum Sq	Mean Sq	*F* value	*p*
TL					
Year	3	656,921.887	218,973.962	111.058	.000
River	3	393,310.528	131,103.509	66.492	.000
Sex	1	860,350.946	860,350.946	436.347	.000
Year:River	8	199,492.923	24,936.615	12.647	.000
Year:Sex	3	9,581.311	3,193.770	1.620	.183
River:Sex	3	36,772.902	12,257.634	6.217	.000
Year:River:Sex	6	39,265.708	6,544.285	3.319	.003
Mass					
Year	3	30.776	10.259	91.847	.000
River	3	23.854	7.951	71.189	.000
Sex	1	38.923	38.923	348.478	.000
Year:River	8	11.978	1.497	13.405	.000
Year:Sex	3	1.025	0.342	3.060	.027
River:Sex	3	2.461	0.820	7.345	.000
Year:River:Sex	6	1.996	0.333	2.979	.007

#### 3.2.2 Size‐at‐age

Our prediction that fish in the southern rivers would be smaller for their age through time was supported, although the reduction in size was small (see Table 5 for posterior means and 95% credible intervals). Overall, fish were larger in the southern than the northern river(s), possibly due to a longer (warmer) growing season leading to larger size (though we do not have thermal data). Over all rivers, fish were 29.4 mm larger contemporaneously than they were historically, and males were 48.2 mm smaller than females on average. Lastly, and the term that tested our hypothesis, fish in the southern rivers were 13.7 mm smaller relative to fish in the north in contemporary relative to historical samples.

**TABLE 5:** Posterior means (PM) and 95% credible intervals (CIs) for walleye size‐at‐age model for total length (in mm)

Parameter	Parameter	PM (mm)	95% CI	Percent of the posterior mass below 0
$\beta_{0}$	Intercept	463.8	418.7–506.4	0%
$\beta_{1}$	Age	10.8	8.6–12.9	0%
$\beta_{2}$	Location	27.8	−9.7–80.4	6%
$\beta_{3}$	History	−29.4	−50.0–−9.3	100%
$\beta_{4}$	Sex	−48.2	−65.6–−32.6	100%
$\beta_{5}$	History x Location	−13.7	−37.1–9.6	87%

Note

Age is the effect of each year on length, location is south versus north, and history is contemporary versus historical.

All parameter estimates passed convergence checks. Each parameter had effective samples sizes > 1,000 and passed the Gelman–Rubin diagnostic test with potential scale reduction factors (PSRF) <1.1, suggesting convergence on a common posterior mode (Gelman et al., 2013).

### 3.3 Population structure

Our prediction that population structure would change over time, possibly including homogenization of structure between southern rivers, was supported for Icon and Perch Rivers in two of the three analyses. Specifically, ADMIXTURE and *F*_ST_ analyses supported this prediction, while DAPC did not. Using both DAPC and ADMIXTURE 3 populations best described the data (*k* = 3,053.162 and CV = 0.421, respectively) (Figure 1), but the difference between CV 2 and CV 4 was small for ADMIXTURE (CV of 2 = 0.423 and CV of 4 = 0.428). In the 3‐population scenario, Icon and Perch grouped as a metapopulation and Chalifour and Takwa Rivers were independent, with 2002/2003 and 2015 samples grouping together for each river. In a 4‐population scenario, many Perch 2003 individuals showed a substantial fraction of loci that were different from the Icon–Perch group. Genetic differentiation by *F_ST_* mirrored what was evident in the *K* = 4 scenario. F_ST_ showed weak differentiation (*p* < .05) between Perch and Icon in 2002/2003 (Table 6), and then merged as a single metapopulation in 2015. At a within‐population level, Chalifour River was differentiated between timepoints, Icon did not diverge between timepoints, Perch diverged marginally between timepoints, and Takwa did not diverge between timepoints. Given that *k* = 3 was identified as the best structure overall, subsequent genetic diversity and Ne analyses were conducted using a metapopulation structure for Icon–Perch, with Chalifour and Takwa identified separately, but years were defined as separate populations for temporal analysis.

**TABLE 6:** *F*_ST_ differentiation for walleye within and between rivers for each year sampled

	Cha 2003	Cha 2015	Ico 2003	Ico 2015	Per 2003	Per 2015	Tak 2003	Tak 2015
Ch 2003	–	*	*	*	*	*	*	*
Ch 2015	0.004	–	*	*	*	*	*	*
Ic 2003	0.02	0.017	–	0.361	*	0.042	*	*
Ic 2015	0.02	0.016	0	–	0.013	0.895	*	*
Pe 2003	0.019	0.019	0.002	0.001	–	0.042	*	*
Pe 2015	0.021	0.017	0.001	0	0.001	–	*	*
Ta 2003	0.053	0.049	0.072	0.07	0.075	0.073	–	0.05
Ta 2015	0.057	0.053	0.077	0.076	0.078	0.078	0.001	–

Note

*F*_ST_ estimates are below the diagonal, and *p*‐values are above the diagonal, with a “*” indicating *p* ≤ .001.

### 3.4 Genetic diversity and Ne

**FIGURE 3:**
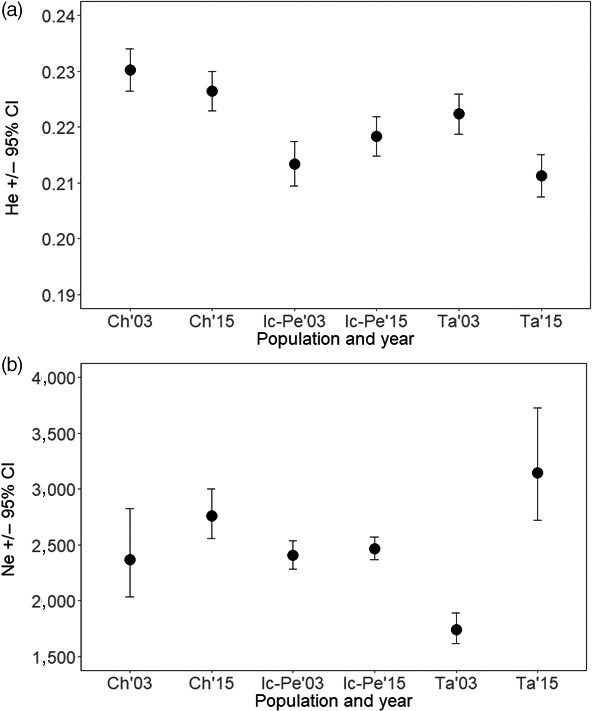
(a) Expected heterozygosity (H_E_) ± 95% CI and (b) effective population size (Ne) ± 95% CI for walleye from each Chalifour (Ch), Icon–Perch (Ic‐Pe), and Takwa (Ta) Rivers in each 2003 and 2015

Our prediction that genetic diversity and Ne would be reduced between historical and contemporary timepoints was not supported. Genetic diversity fell within a tight range for all populations over all years, ranging from 0.21 to 0.23 (Figure 3a). Confidence Intervals (CIs) overlapped between timepoints for H_E_ in Chalifour and Icon–Perch, and there was a 4.9% loss in H_E_ in Takwa, though the reduced H_E_ still fell within the range of southern populations. Point estimates of Ne ranged from 1741 to 3,146 individuals across all populations, with the lowest and highest values being in the northern population. Ne CIs also overlapped between timepoints in Chalifour and Icon–Perch Rivers, and the data suggested a doubling in Ne over time in Takwa River (Figure 3b). There were likely insufficient samples sequenced per population to accurately detect a difference between time periods in populations consisting of thousands of individuals, and reliable detection of changes to Ne often requires ~ ten generations to have passed (Nunziata & Weisrock, 2018; Waples & Do, 2010); however, these results demonstrate that all populations remained large (i.e., Ne in the thousands).

### 3.5 Signatures of selection

**FIGURE 4:**
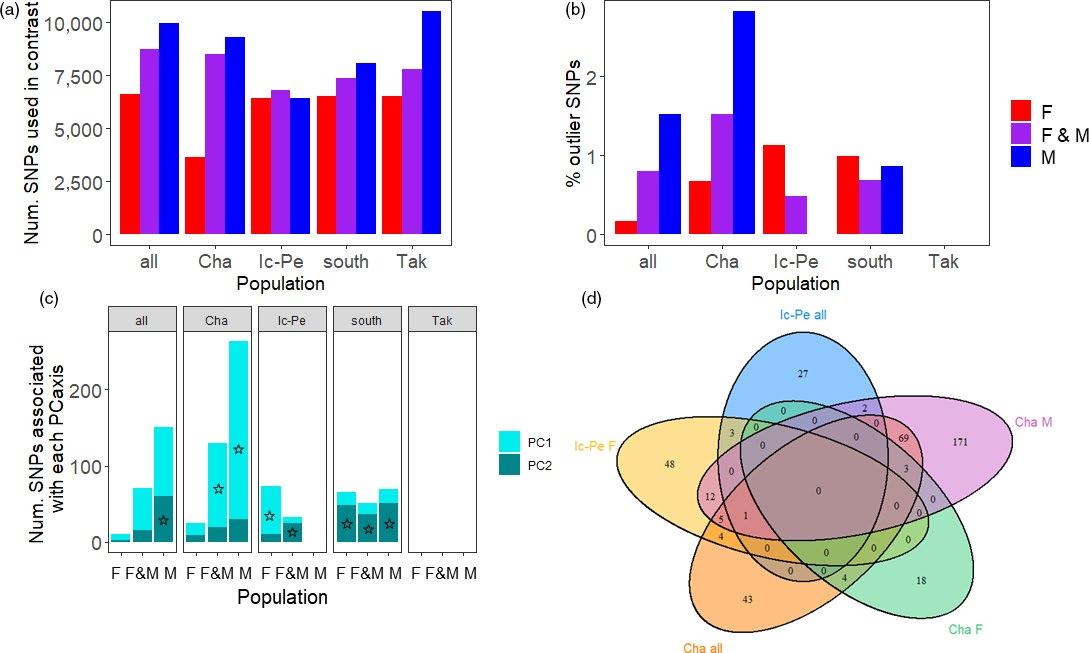
(a) Total number of SNPs used in each between‐year contrast (i.e., the number of SNPs used was unique for each sex and population). (b) Percentage of SNPs that were outliers in each pairwise contrast. (c) Number of outlier SNPs associated with each PC axis. The star (☆) denotes which PC axis separated years. Where there is no star on a bar, population structure between years was not maintained by outlier loci. See Table S8 and Figures S2–S16 for further explanations and detailed descriptions of how outliers on each PC axis maintained the observed population structure. Where no bars are shown (i.e., for Takwa and Icon–Perch M in % outlier SNPs and SNPs associated with each PC axis), there was no population structure and thus were no outlier loci between years. (d) Outlier loci overlap between the southern populations

Our prediction that signatures of selection would be present between historic and contemporary timepoints within southern rivers but not the northern river, with putatively selected loci related to growth, body size, and/or maturation, was supported. Eleven to 263 loci were outliers (0.17%–2.83%) depending on the scenario tested (Figure 4a‐b). Outliers were found in the global PCA that included all rivers and both timepoints, for each F/M together, and for F & M individually. Removing these outliers did not change the population structure when all rivers (i.e., including Takwa) and years were assessed. On the contrary, when the southern rivers were analyzed as a unit (i.e., the south historic versus contemporary) and on their own (i.e., each Chalifour and Icon–Perch historic versus contemporary years), for F/M combined and for each F & M separately, population structure existed between the two timepoints and removing outlier loci usually collapsed the population structure. Further, there was no population structure between timepoints in Takwa (and thus no outlier loci) (Figure 4b, Table S8, and Figures S2–S16). In sum, while there were outliers separating north from south, outlier SNPs disproportionately contributed to the PCs between years in the southern subset. In addition, the greatest proportion of outlier SNPs found in each historic/contemporary PCA in the south maintained population structure between years (Figure 4c). Lastly, parallel outliers existed between the southern rivers (Figure 4d); of note, more outliers were in common between Icon–Perch F and Chalifour *M* (18 outliers) than between Icon–Perch F and Chalifour *F* (0) or Chalifour M and *F* (3).

Sex‐specific analyses provided greater resolution than with sexes combined (Figures 4c and d). For example, outlier SNPs maintained population structure between timepoints in more of the southern rivers in sex‐specific rather than combined analyses (Table S8, Figures S2–S7). In addition, there was no population structure in Icon–Perch males, but there was in females (Figure 4c and Figures S12–S13). Lastly, the number of outliers in common between rivers differed between the sexes (Figure 4d), though this may be due in part to the number of individuals sequenced.

Blasts were conducted for southern (F/M combined and separately), Chalifour (F/M combined and separately), and Icon–Perch *F* (no outliers were found for Icon–Perch M or Takwa) Rivers. Southern F/M combined and M had no blast hits. Otherwise, between 2 and 6 alleles were annotated for each blast. Because annotations were completed against all mapped Metazoa, at level 2 go annotation, functional annotations included many different biological processes, molecular functions, and cellular components. Three relevant processes indicated were growth, metabolism, and developmental process (Table S9).

## 4 DISCUSSION

Our results suggest that nascent genetic changes associated with harvesting can arise in wild fish populations within a 1–2.5 generation period, a shorter timescale than previously observed in other fisheries. Concurrent with reductions in body size within a 15‐year period (2002/2003–2017) (Table 3, Figure 2), we detected a small reduction in size‐at‐age (Table 5), emerging genomic changes evidenced by changing genomic population structure (Figure 1, Table 6) and putative signatures of selection within rivers (Figure 4), with sexes both combined and separately. These changes were present in the southern rivers most impacted by increased fishing pressure by Cree and non‐Cree fishers alike (Tables 2 and Figure S2), and not in the northern river where there were fewer boats and fishers. Importantly, not only is fishing pressure greatest in the south, but also southern fish from the affected spawning runs remain close to those spawning runs in the summer mixed‐population fishery (Dupont et al., 2007). Furthermore, all study populations had large Ne, making it unlikely that genetic drift was responsible for the phenotypic or genetic changes observed in the southern rivers. The difference between neutral and putatively adaptive genomic results illustrates the capacity for genetically large populations in nature to rapidly respond to changing selection pressures.

Congruent with IK, Western scientific methods showed consistent reductions in body size (or trends indicating such reductions) between 2002/2003 and each of 2015–2017 within all southern rivers, except for in Perch River males (Figure 2); moreover, size‐at‐age was reduced in the south over time (Table 5). In fact, size reductions in all southern rivers may be underestimates of the true change.Namely, 2016 monitoring was largely collaborative; approximately 48% of all sampled walleye (216 of 446) were harvested and donated by fishers. Donated 2016 walleye were 639 g (stderr ± 21 mm, females) and 424 mm (stderr ± 4 mm, males) on average compared to 603 g (stderr ± 20 g, females) and 410 mm (stderr ± 4 mm, males) for our caught and released 2016 walleye (note that sampling was not collaborative in this way in 2002/2003). Lastly, given that Perch River males were smaller than females, it is less likely that males would be subject to size‐selective harvesting (to explain their lack of changes to body size over time). In sum, these results are consistent with the idea that fishers often target larger fish, and this type of size‐selective harvesting has been documented to lead to the evolution of smaller body size (Heino et al., 2015; Hutchings, 2005; Swain, Sinclair, & Hanson, 1993, 2007).

Genomic change occurred between timepoints in the southern rivers but not in the northern river. Population structure was homogenized over time between Icon and Perch Rivers, and Chalifour and Perch Rivers were differentiated between timepoints at neutral loci. In addition, parallel outlier loci were detected between timepoints in all southern rivers (Figure 4c), and exploratory analysis of outlier loci within southern rivers (Figure 4, Table S8) revealed relevant biological functions associated with a small number of those loci (Table S9). However, genomic changes were clearly nascent. Differences between the preferred population structures in ADMIXTURE were small (Figure 1). F_ST_ within Chalifour and Perch Rivers between years and between Icon and Perch 2015 were small (Table 6), and scree plots for outlier locus detection showed weak structure in two cases (Figures S9 and S12). From a pragmatic perspective, we could have focused on *k* = 2 or 3 as the only possible population structure, rejecting homogenization of population structure in Icon–Perch and differentiation at neutral loci. Even disregarding the neutral genomic change, however, trends in outlier loci were generally consistent when sexes were analyzed together and separately; although congruent with reductions in body size between 2002/03 and 2015 in Perch River, genetic structure was present between timepoints in Icon–Perch females but not males (Figure 4).

Signatures of selection (i.e., Atlantic cod (Therkildsen et al., 2013)) and changes in population structure (Atlantic cod (Hutchinson et al., 2003)) have been associated with harvest in other studies on wild fish populations, and other harvested species such as red deer (Frantz, Hamann, & Klein, 2017, 2008; Nussey et al., 2005). Allen et al. (2017) observed changes in H_E_in walleye that we did not see here, but 4–8 generations passed in their study compared to 1–2.5 in our study. Decreases in genetic diversity have also been associated with stock declines (biomass) over differing timescales and varying fishing pressures (Hauser, Adcock, Smith, Ramírez, & Carvalho, 2002; Hutchinson et al., 2003; Smith, Francis, & McVeagh, 1991) or with fishing pressure, but no phenotypic or biomass data were presented (Jones, McParland, Hutchings, & Danzmann, 2001). Moreover, the simulation model of Audzijonyte, Kuparinen, Gorton, and Fulton (2013) showed that even gradual length reductions of 0.1%/yr in five harvested fish species could affect species interactions, as well as biomasses and yields by 1%–35% over 50 years.

While our data suggest rapid genetic changes to population structure and genetically based phenotypes associated with harvest, we do not have a direct link with harvest, and alternative explanations must be explored. We firstly note that the observed reduction in size‐at‐age in the south was small relative to the overall change in body size between historical and contemporary samples, and may be due to a difference in aging structures used (historical using opercula, contemporary using otoliths) (Faust & Scholten, 2017, 2008). However, ages calculated using opercula and otoliths have been highly correlated in walleye (Geisler, 2012), and opercula have been validated to the age of 16 in walleye (94% of aged fish in Mistassini were < 16) (Faust & Scholten, 2017, 2008).

Under variable recruitment (Bozek et al., 2011, 2015; Hansen, Bozek, Newby, Newman, & Staggs, 1998), fish captured in 2002 and 2003 could also represent distinct, strong year classes, biasing estimates of mean size and contributing to temporal genetic differences. However, there was no genetic differentiation within rivers between 2002 and 2003 (Dupont et al., 2007), walleye from the northern river (Takwa) had consistent body sizes in all four years sampled, and we found a significant reduction in size‐at‐age in the southern rivers.

Another alternative could be that the large body size changes in southern rivers are entirely plastic responses due to changes in the environment or a habitat shift unrelated to fishing. However, climate change is expected to warm the Mistassini Lake region; as a cold oligotrophic lake, Mistassini is not ideal habitat for walleye, which prefer mesotrophic lakes (Kitchell et al., 1977; Niemuth, Churchhill, & Wirth, 1972). Climate warming is expected to increase the growing season length for walleye, and thus in the absence of fishing, an increase in body size is a more likely response with climate warming than a decrease. Regarding plasticity, growing degree day (GDD) was shown to account for 96% of the variation in length of immature walleye over 416 populations in Ontario and Quebec, though variation in growth associated with food availability was also evident (Neuheimer & Taggart, 2007; Venturelli, Lester, Marshall, & Shuter, 2010). Thus, although it is unlikely that all observed changes are due to selection, smaller body size at spawning and smaller size‐at‐age could indicate that fish are selectively growing slower (Enberg et al., 2012). Of course, we do acknowledge that climate warming/change might alternatively reduce walleye growth by affecting their prey species disproportionately more in the southern part of Mistassini Lake where southern populations forage.

Further, we estimated the selective pressure required to generate the observed changes in body size within 1–2.5 generations to assess whether they were biologically plausible using the breeder's equation (*R = h^2^S*, where *R = *response to selection, *h^2^ = *heritability,* S* = selection differential). Using averages for 11‐year‐old walleye in the south for each 2002 (515 mm) and 2015 (424 mm) (a 13‐year interval), *R* = −7 mm per year. Given realistic *h^2^* estimates (0.3) (Law, 2000; Nussle, Bornand, & Wedekind, 2009), if observed changes were entirely due to selection, *S* would need to be −23 mm per year. Our Bayesian model held all variables constant when assessing the size change associated with harvest, and in so doing found that the size change attributed to selection was smaller than what is shown here. Thus, it is very likely that some of the observed body size change was plastic and/or due to stochasticity in addition to selection pressure.

Lastly, alternative explanations for the nascent genomic change evident in Icon–Perch could also include sampling bias, spatial movement, or increased gene flow. If sampling bias was present, Perch 2003 individuals could have been from a different population, but 2002/03 samples were genetically indistinct within rivers (Dupont et al., 2007), and Perch 2003 grouped with 2015 samples by DAPC. Alternatively, Perch 2003 individuals that were different historically could have moved to a different spawning location in later years (Bigrigg, 2008), though Mistassini Lake populations generally have strong spawning site fidelity (Dupont et al., 2007). Another possibility is that individuals from Icon River could be using Perch River to spawn much more now than historically, either replacing genotypes that have been fished out or increasing gene flow substantially (Allendorf et al., 2008). Although the observed neutral and putatively selective genomic changes in Icon–Perch are rapid, they are not without precedent (3 generations or less; Chebib et al., 2016; van Wijk et al., 2013), and even though these are genetically large populations (Figure 3a), rapid adaptation is possible via soft sweeps (Hermisson, 2005; Messer & Petrov, 2013). In sum, nascent genomic change occurred within a 12‐year period within the southern most‐harvested rivers (genomic samples were 2003 and 2015), which represents 1–2.5 generations maximum.

### 4.1 Conclusions and management implications

We have presented coupled IK, phenotypic, and genetic evidence consistent with genetic changes associated with harvesting within 1–2.5 generations in wild walleye populations. Links with fishing pressure are not conclusive, but this study sets a precedent for the time frame needed for investigating concerns regarding harvest‐induced evolution in fisheries. Furthermore, sex‐specific dynamics for both body size and genomics herein highlight the importance of collecting sex‐specific data; if we had looked at both sexes together, the stronger signal in females (i.e., possible changes in fecundity) would likely have been muted, providing inaccurate information for fisheries managers.

Our study illustrates the benefits of integrating life history and genomic methods for conservation in order to understand the factors affecting population change (Bernatchez et al., 2017), of interweaving these with IK, and of iterative population monitoring practices (Flanagan, Forester, Latch, Aitken, & Hoban, 2018); that is, this study would not have been possible without the historic data. Considerations for Cree management could include that observed phenotypic and genetic changes may cause reduced productivity (Allendorf et al., 2008; Hutchings, 2005) and that genomic changes are clearly nascent here. Depending on the severity of harvest (which is not precisely known in this case) and life history (Audzijonyte & Kuparinen, 2016), fisheries‐induced changes may be reversed in 9 generations (Conover, Duffy, & Hice, 2009 or less (Feiner et al., 2015) if fishing is halted; the ability of populations to recover depends on their trophic position, age‐specific fecundity, and survival at each life stage (Audzijonyte & Kuparinen, 2016) (Box 1).

BOX 1 **Reflections on using Indigenous knowledge (IK) together with Western scientific methods (WSM) toward resource conservation**

One million species are currently threatened by extinction globally, but nature is declining less in lands managed by Indigenous peoples (IPBES, 2019). Land users have a tremendous amount of knowledge about the resources and land they use (Berkes, Colding, & Folke, 2000). According to the 2019 Intergovernmental Science‐Policy Platform on Biodiversity and Ecosystem Services (IPBES) report, novel solutions are needed to reverse the trajectory of species and environmental decline. A key mandate of two conservation agencies in Canada (Parks Canada (part of Environment and Climate Change Canada), and the Department of Fisheries and Oceans) is to interweave IK into management (DFO, 2019b; Parks Canada, 2019). However, in current monitoring frameworks, IK is not mentioned (DFO, 2019a; Parks Canada, 2018). Therefore, these frameworks need to be developed. Published studies have shown how IK and WSM can provide complimentary or congruent results for population monitoring (Fraser, Calvert, Bernatchez, & Coon, 2013; Fraser et al., 2013; Polfus, Heinemeyer, & Hebblewhite, 2014). In our work, IK acted like an early warning, providing the basis for the study, and then also providing us with important information regarding potential causes of change (i.e., fishing pressure). We could then use WSM to uncover a more detailed understanding of what was happening in the system to inform management actions.

I (Dylan) am fortunate to have worked with the Cree Nation of Mistissini since 2000 when I started graduate studies in Louis Bernatchez’ Lab. I have a long‐standing interest in conservation biology and have long admired the incredible knowledge that Cree fishers have of the fish found on their traditional territories. Like many Indigenous groups in northern Canada, the Cree seek a balance between maintaining their own cultural traditions, protecting the environment for future generations, and developing economically, using the best available information. Owing to past colonialism, a framework that involves active collaborations with Indigenous peoples is essential for improving resource conservation in northern Canada and for deriving the full socio‐economic benefits of fisheries and other natural resources. The integration of IK and WSM, as two different but complementary ways of knowing, is a critical step toward fostering local resource management and empowering local communities to make effective resource conservation decisions.

I (Ella) began working in the same system as Dylan in 2016 as a postdoc in his laboratory. My career goal is to work in areas related to biodiversity conservation, and I believe strongly that any conservation efforts that biologists are involved in should be in concert with stewards of the land. Stewards include Indigenous groups with a long history on the land, or also other hunters, fishers, or farmers who have a long history using a given resource. In ecology, understanding results is often very rooted in recognizing the idiosyncrasies of a system, which stewards often know better than scientists. I did not learn how to weave knowledge types during my earlier training, and the ability to work with the Cree Nation of Mistissini and learn how to interweave IK with WSM toward conservation was a central motivation for my wanting to join Dylan’s lab.

In my (Pamela) academic and work experience thus far, one of the perspectives I recognized and was able to relate in some way was the unique knowledge and connection Indigenous people have to the environment. For hundreds of years, their survival depended on a harmonious relationship with their surrounding land, water, wildlife, etc. The Cree skills gained have been passed on from one generation to another, and in most cases, conservation measures are created for the purpose of continuing to transfer this knowledge. Being raised in my Cree community of Mistissini, and working in the field of environment for the past six years, has allowed me to understand the expertise our land users and community members have because of this established relationship. I believe the use of Indigenous knowledge and Western science methods allows for a more detailed understanding of the research being studied, though further work needs to be done to define how the two knowledge types are or can be applied. These efforts are currently ongoing with the fish studies we (in Mistissini) have conducted with Concordia University over the years. It is my objective to ensure the existing Indigenous knowledge, more specifically the knowledge from the Cree people of Mistissini, is respected and incorporated in research and decision‐making regarding environmental management.

## CONFLICT OF INTEREST

The authors declare no competing interests.

## Supporting information

Supplementary Material

## Data Availability

Supporting data are available on Dryad: https://doi.org/10.5061/dryad.5tb2rbp1z (Bowles, Marin, Mogensen, MacLeod, & Fraser, 2020).
