# Supplementary material for: Size reductions and genomic changes within two generations in wild walleye populations: associated with harvest?
Source: Evol Appl. 2020 May 25;13(6):1128–44. doi: 10.1111/eva.12987 (PMC7359826; doi:10.1111/eva.12987)
Supplement: Supplementary file 1 — Supplementary Material [file EVA-13-1128-s001.docx]

**Supporting information**

**Supporting Tables**

[Table S1 Questions asked of 17 Cree elders and fishers who have >25 years of walleye fishing experience on Mistassini Lake. Only a subset of these questions were used in this manuscript, but the complete list of questions is included to indicate the depth and bredth of knowledge gathered. 3](#_Toc38381497)

[Table S2 Demographic trends for the population in Mistissini and documented number of fish harvested by non-Cree fishers. The fish caught by non-Cree fishers were all in the southern half of lake below the 51st parallel (num fish non-Cree). Aboriginal affairs branch, Ministry of health and social services, Government of Quebec (GC). Ministry of Forests, Wildlife and Parks, Government of Quebec (MFFP). Cree Nation of Mistissini (CNM), Statistics Canada (GQ). 3](#_Toc38381498)

[Table S3 Total length (TL) lsmeans with the lower (LCI) and upper (UCI) 95% confidence intervals. 4](#_Toc38381499)

[Table S4 Total length contrasts. Where insufficient data existed to make a contrast, NA is shown. 5](#_Toc38381500)

[Table S5 Mass lsmean with lower (LCI) and upper (UCI) 95% confidence interval 6](#_Toc38381501)

[Table S6 Mass contrasts. Where insufficient data existed to make a contrast, NA is indicated. 7](#_Toc38381502)

[Table S7 Number of individuals successfully sequenced and retained after all filtering (n), number of private alleles (private), number of SNPs (SNPs), percent polymorphic loci (% poly), expected heterozygosity (HE), observed heterozygosity (HO), pi (π). 10](#_Toc38381503)

[Table S8 Outlier loci, PC axis on which they are relevant, and explanation of the population structure explained by each PC axis for historic-contemporary contrasts for each population separately and all populations combined. Figures S2 to S16 illustrate the results described in this table. 10](#_Toc38381504)

[Table S9 Outlier loci blasted, mapped and annotated using blast2go. Annotation for gene ontology level 2. Biological process (BP), Molecular function (MF) and Cellular component (CC). 11](#_Toc38381505)

**Supporting Figures**

[Figure S1 Sex-bias at sampling sites, where blue = males, red = females and grey = unknowns in 2002-03, 2015, 2016 and 2017. Also shown are the total captured walleye in each river (n). 9](#_Toc38381481)

[Figure S2 All populations, all years, m and f combined, k=2. Top: scree panels for selecting best k. Bottom left, with outliers left in. Bottom right, excluding outliers. Result: PC1 separates the north from the south, and also Icon03/15Perch15 a bit, and the second PC separates Chalifour from the other pops. Shown in the SNP list that associates SNPs with PCs below, the vast majority of outliers are associated with PC1 (15 to PC2). However, removing outliers does not change the structure in a meaningful way. 13](#_Toc38381482)

[Figure S3 All populations and years, f, k=2. Top panels are scree plots for determining the best k. Bottom panels are: (left) including outliers, and (right) excluding outliers. Result: PC1 separates north and south, and maybe Chali03/15 and Pe03 a little bit from the Ic03,15,Pe15 group. PC2 separates Ch03 from Ch15, and also both Chalifour years from the other southern pops. Result looks similar to when M and F are combined, except that PC2 also separates Ch03 and 15. Removing outliers has no meaningful affect on the population structure. number of pops that I required loci to be present in for the full dataset is 6/8. 16](#_Toc38381483)

[Figure S4 All populations and years, m, k=2. Top panels are scree plots used to determine best k. Bottom panels are: (left) including outliers, and (right) excluding outliers. Result: PC1 separates north from south, and also Pe03 and the Chalifours. PC2 separates Cha03 and 15 from the other pops, Pe03 from the other pops, and Ch03 from 15. Removing outliers removes the structure between Ch03 and 15. 18](#_Toc38381484)

[Figure S5 Southern rivers, m & f combined, k=2. Top right panel is with outliers included and bottom right panel is outliers excluded. Conclusions: structure is sufficient to detect outliers. PCI separates Ch from other pops, PC2 separates Ch 03 from 15 and Pe03. Removing outliers removes the structure. 23](#_Toc38381485)

[Figure S6 southern rivers, f, k=2. Top panels are the scree plots used to find the best k. Bottom panels are: (left) including outliers and (right) excluding outliers. Result: PC1 separates Chalifour from the other sites and PC2 separates Pe03 and Cha03 from the other sites. Removing outliers collapses PC2 structure, collapsing Pe03 into the other southern rivers, and Ch03 and 15. This result is the inverse of the result when both sexes are analyzed together. 25](#_Toc38381486)

[Figure S7 south, m, k = 2. Top panels are scree plots used to select the best k. Bottom panels are: (left) including outliers, and (right) excluding outliers. Result: PC1 separates Chalifours from the other sites and Pe03 from Ic. PC2 seaprates Cha03 from 15. Removing outliers collapses Cha 03 and 15, and also collapses Pe03 completely into the other sites. 28](#_Toc38381487)

[Figure S8 Chalifour, m and f combined. Upper left is scree plot showing that k=2 is best. Top right is including outliers, bottom right is excluding outliers. Outliers play a role in generating population structure within this river between years, shown by changes in population structure when outliers are excluded. 31](#_Toc38381488)

[Figure S9 Chalifour f, k = 2. Top, with outliers, bottom, without outliers. There is sufficient structure to determine outliers. Based on the number of individuals it isn’t totally clear that the outliers are maintaining the structure present. Populations might overlap more when outliers are removed. 35](#_Toc38381489)

[Figure S10 Chalifour m, k = 2. Top left panel is the scree plot showing best k. Top right is including outliers and lower right is excluding outliers. Result: PC1 separates the timepoints. 37](#_Toc38381490)

[Figure S11 Ic-Pe, m and f combined. Top left is the scree plot showing that k = 5 looks best. However, contribution of PC3 to population structure was tested, and it does not change the conclusion. Thus, k=2 was chosen. Top right is with outlier loci included, and bottom right is with outlier loci excluded. Conclusion: although structure is relatively weak within Ic-Pe between years, there is structure, and outlier loci contribute to this structure. Pe03 separates out on PC2. In SNP list associated with PCs below, you can see that 8 SNPs are associated with this PC. 44](#_Toc38381491)

[Figure S12 Ic-Pe f, k=2. Top panels are scree plots used to determine best k. Bottom plots are (right) with outlier loci included, and (left) excluding outlier loci. Result: PC1 separates Pe03 from the other populations. 46](#_Toc38381492)

[Figure S13 Ic-Pe m, k = 4. Top panels are scree plots showing that there is insufficient structure to find a best k. Bottom panel is including outliers. Both k = 4 and k = 6 were tested with the same result. 49](#_Toc38381493)

[Figure S14 Takwa, m and f combined k=2, The top panel is scree plots showing the best k. Bottom panel is with outliers included, and shows that there is no structure between years. The structure seen in the scree plot is likely due to those outlier individuals. 50](#_Toc38381494)

[Figure S15 Takwa River, f, k=2. Top panels are scree plots used to find the best k. Bottom panel is including outliers. Result: There is no structure between years within Takwa River. Outliers cannot be assessed. 51](#_Toc38381495)

[Figure S16 Takwa m, k=3. Top left panel showing scree plot used to find best k. Top right panel is including outliers. Bottom right panel is excluding outliers. Result: there is no population structure between years, and thus no outliers can be found. 52](#_Toc38381496)

*Indigenous Knowledge*

Table S1 Questions asked of 17 Cree elders and fishers who have >25 years of walleye fishing experience on Mistassini Lake. Only a subset of these questions were used in this manuscript, but the complete list of questions is included to indicate the depth and bredth of knowledge gathered.

| **Subject** | **General Question** |
| --- | --- |
| General Information | How old are you? |
|  | How many years have you been fishing for walleye (was always interpreted as how many years the informant had been fishing)? |
| Ecology | Where do you fish for walleye? |
|  | At what depth do you fish for walleye? |
|  | When/at what season do you fish for walleye? |
|  | How do you capture walleye (what fishing gear do you use)? |
|  | How big, on average, are the walleye that you capture now (used tape measure as a guide)? |
|  | Do the walleye look the same throughout the lake? |
|  | Where do walleye spawn? |
|  | When do walleye spawn? |
|  | Do you fish for walleye when they spawn? |
| Conservation | Has the location where you fish for walleye changed since you started fishing, or over the last 40, 30, 20, 10, 5 years? |
|  | Has the depth at which you fish for walleye changed since you started fishing, or over the last 40, 30, 20, 10, 5 years? |
|  | Has the time when you fish for walleye changed since you started fishing, or over the last 40, 30, 20, 10, 5 years? |
|  | Has the way you capture walleye (i.e., fishing gear) changed since you started fishing, or over the last 40, 30, 20, 10, 5 years? |
|  | Has the size of walleye changed since you started fishing, or over the last 40, 30, 20, 10, 5 years? |
|  | If walleye look the same or different throughout the lake, has this changed since you started fishing, or over the last 40, 30, 20, 10, 5 years? |
|  | Has where walleye spawn changed since you started fishing, or over the last 40, 30, 20, 10, 5 years? |
|  | Have the number of walleye changed since you started fishing, or over the last 40, 30, 20, 10, 5 years? |
|  | Do you have any concerns about the overall health of the walleye throughout the lake? |
|  | If you do have concerns about the overall health of walleye throughout the lake, what factors might be contributing to this decline over the last 40, 30, 20, 10, 5 years? |
|  | If you do have concerns about the overall health of the walleye throughout the lake, what do you think could be a solution to this decline? |
|  | What do you think could be done to help protect the walleye for future generations? |

*Demography*

Table S2 Demographic trends for the population in Mistissini and documented number of fish harvested by non-Cree fishers. The fish caught by non-Cree fishers were all in the southern half of lake below the 51st parallel (num fish non-Cree). Aboriginal affairs branch, Ministry of health and social services, Government of Quebec (GC). Ministry of Forests, Wildlife and Parks, Government of Quebec (MFFP). Cree Nation of Mistissini (CNM), Statistics Canada (GQ).

|  | **1997** | **Source** | **2003** | **Source** | **2011** | **Source** | **2015** | **Source** | **2016** | **Source** |
| --- | --- | --- | --- | --- | --- | --- | --- | --- | --- | --- |
| **Population** | 2479 | GQ | 2863 | GQ | 3469 | GQ | 3654 | GQ | 3724 | GQ |
| **Num. of households** | 424 | CNM | 556 | CNM | - | - | - | - | 800 | GC |
| **Num. fish non-Cree** | 5800 | MFFP | 8916 | MFFP | 5572 | MFFP | 5725 | MFFP |  |  |

*Life History*

Body size

Table S3 Total length (TL) lsmeans with the lower (LCI) and upper (UCI) 95% confidence intervals.

| **River** | **Year** | **Sex** | **lsmean** | **SE** | **df** | **LCI** | **UCI** |
| --- | --- | --- | --- | --- | --- | --- | --- |
| Chalifour | Y2002/03 | F | 505.889 | 14.801 | 1486 | 476.855 | 534.923 |
| Chalifour | Y2015 | F | 431.714 | 11.867 | 1486 | 408.436 | 454.993 |
| Chalifour | Y2016 | F | 472.414 | 8.246 | 1486 | 456.240 | 488.588 |
| Chalifour | Y2017 | F | 460.167 | 12.818 | 1486 | 435.023 | 485.311 |
| Chalifour | Y2002/03 | M | 465.111 | 4.463 | 1486 | 456.357 | 473.865 |
| Chalifour | Y2015 | M | 416.581 | 4.105 | 1486 | 408.529 | 424.634 |
| Chalifour | Y2016 | M | 429.606 | 3.865 | 1486 | 422.025 | 437.187 |
| Chalifour | Y2017 | M | 410.333 | 4.532 | 1486 | 401.444 | 419.223 |
| Icon | Y2002/03 | F | 549.033 | 8.107 | 1486 | 533.131 | 564.936 |
| Icon | Y2015 | F | 435.125 | 15.699 | 1486 | 404.330 | 465.920 |
| Icon | Y2016 | F | 467.692 | 12.315 | 1486 | 443.535 | 491.850 |
| Icon | Y2002/03 | M | 458.636 | 6.694 | 1486 | 445.505 | 471.767 |
| Icon | Y2015 | M | 393.000 | 4.333 | 1486 | 384.500 | 401.500 |
| Icon | Y2016 | M | 394.161 | 3.567 | 1486 | 387.165 | 401.157 |
| Icon | Y2017 | M | 376.579 | 7.203 | 1486 | 362.449 | 390.709 |
| Perch | Y2002/03 | F | 498.892 | 7.300 | 1486 | 484.573 | 513.211 |
| Perch | Y2015 | F | 441.077 | 12.315 | 1486 | 416.919 | 465.234 |
| Perch | Y2016 | F | 456.360 | 8.881 | 1486 | 438.940 | 473.780 |
| Perch | Y2002/03 | M | 421.899 | 4.707 | 1486 | 412.666 | 431.132 |
| Perch | Y2015 | M | 413.706 | 7.615 | 1486 | 398.768 | 428.644 |
| Perch | Y2016 | M | 392.538 | 5.028 | 1486 | 382.676 | 402.401 |
| Perch | Y2017 | M | 399.667 | 12.818 | 1486 | 374.523 | 424.811 |
| Takwa | Y2002/03 | F | 501.511 | 6.619 | 1486 | 488.527 | 514.495 |
| Takwa | Y2015 | F | 509.400 | 6.280 | 1486 | 497.082 | 521.718 |
| Takwa | Y2017 | F | 496.143 | 9.690 | 1486 | 477.136 | 515.150 |
| Takwa | Y2002/03 | M | 448.935 | 6.547 | 1486 | 436.092 | 461.777 |
| Takwa | Y2015 | M | 433.254 | 4.159 | 1486 | 425.097 | 441.412 |
| Takwa | Y2017 | M | 433.918 | 6.343 | 1486 | 421.475 | 446.361 |

Table S4 Total length contrasts. Where insufficient data existed to make a contrast, NA is shown.

| **River** | **Contrast** | | **Sex** | | **estimate** | | **SE** | | **df** | | ***t.ratio*** | | ***P*** |
| --- | --- | --- | --- | --- | --- | --- | --- | --- | --- | --- | --- | --- | --- |
| Chalifour | Y2002/03 - Y2015 | | F | | 74.175 | | 18.971 | | 1486 | | 3.910 | | 0.003 |
| Chalifour | Y2002/03 - Y2016 | | F | | 33.475 | | 16.943 | | 1486 | | 1.976 | | 0.232 |
| Chalifour | Y2002/03 - Y2017 | | F | | 45.722 | | 19.580 | | 1486 | | 2.335 | | 0.157 |
| Chalifour | Y2015 - Y2016 | | F | | -40.700 | | 14.451 | | 1486 | | -2.816 | | 0.067 |
| Chalifour | Y2015 - Y2017 | | F | | -28.452 | | 17.468 | | 1486 | | -1.629 | | 0.379 |
| Chalifour | Y2016 - Y2017 | | F | | 12.247 | | 15.241 | | 1486 | | 0.804 | | 0.954 |
| Chalifour | Y2002/03 - Y2015 | | M | | 48.530 | | 6.064 | | 1486 | | 8.003 | | 0.000 |
| Chalifour | Y2002/03 - Y2016 | | M | | 35.505 | | 5.904 | | 1486 | | 6.014 | | 0.000 |
| Chalifour | Y2002/03 - Y2017 | | M | | 54.778 | | 6.360 | | 1486 | | 8.612 | | 0.000 |
| Chalifour | Y2015 - Y2016 | | M | | -13.025 | | 5.638 | | 1486 | | -2.310 | | 0.108 |
| Chalifour | Y2015 - Y2017 | | M | | 6.248 | | 6.115 | | 1486 | | 1.022 | | 0.756 |
| Chalifour | Y2016 - Y2017 | | M | | 19.273 | | 5.956 | | 1486 | | 3.236 | | 0.009 |
| Icon | Y2002/03 - Y2015 | | F | | 113.908 | | 17.669 | | 1486 | | 6.447 | | 0.000 |
| Icon | Y2002/03 - Y2016 | | F | | 81.341 | | 14.744 | | 1486 | | 5.517 | | 0.000 |
| Icon | Y2002/03 - Y2017 | | F | | NA | | NA | | NA | | NA | | NA |
| Icon | Y2015 - Y2016 | | F | | -32.567 | | 19.953 | | 1486 | | -1.632 | | 0.577 |
| Icon | Y2015 - Y2017 | | F | | NA | | NA | | NA | | NA | | NA |
| Icon | Y2016 - Y2017 | | F | | NA | | NA | | NA | | NA | | NA |
| Icon | Y2002/03 - Y2015 | | M | | 65.636 | | 7.974 | | 1486 | | 8.231 | | 0.000 |
| Icon | Y2002/03 - Y2016 | | M | | 64.475 | | 7.585 | | 1486 | | 8.500 | | 0.000 |
| Icon | Y2002/03 - Y2017 | | M | | 82.057 | | 9.834 | | 1486 | | 8.345 | | 0.000 |
| Icon | Y2015 - Y2016 | | M | | -1.161 | | 5.612 | | 1486 | | -0.207 | | 1.000 |
| Icon | Y2015 - Y2017 | | M | | 16.421 | | 8.406 | | 1486 | | 1.953 | | 0.207 |
| Icon | Y2016 - Y2017 | | M | | 17.582 | | 8.038 | | 1486 | | 2.187 | | 0.163 |
| Perch | Y2002/03 - Y2015 | | F | | 57.815 | | 14.316 | | 1486 | | 4.038 | | 0.002 |
| Perch | Y2002/03 - Y2016 | | F | | 42.532 | | 11.496 | | 1486 | | 3.700 | | 0.004 |
| Perch | Y2002/03 - Y2017 | | F | | NA | | NA | | NA | | NA | | NA |
| Perch | Y2015 - Y2016 | | F | | -15.283 | | 15.184 | | 1486 | | -1.007 | | 1.000 |
| Perch | Y2015 - Y2017 | | F | | NA | | NA | | NA | | NA | | NA |
| Perch | Y2016 - Y2017 | | F | | NA | | NA | | NA | | NA | | NA |
| Perch | Y2002/03 - Y2015 | | M | | 8.193 | | 8.952 | | 1486 | | 0.915 | | 0.954 |
| Perch | Y2002/03 - Y2016 | | M | | 29.360 | | 6.887 | | 1486 | | 4.263 | | 0.001 |
| Perch | Y2002/03 - Y2017 | | M | | 22.232 | | 13.655 | | 1486 | | 1.628 | | 0.577 |
| Perch | Y2015 - Y2016 | | M | | 21.167 | | 9.125 | | 1486 | | 2.320 | | 0.207 |
| Perch | Y2015 - Y2017 | | M | | 14.039 | | 14.910 | | 1486 | | 0.942 | | 0.954 |
| Perch | Y2016 - Y2017 | | M | | -7.128 | | 13.769 | | 1486 | | -0.518 | | 1.000 |
| Takwa | Y2002/03 - Y2015 | | F | | -7.889 | | 9.124 | | 1486 | | -0.865 | | 1.000 |
| Takwa | Y2002/03 - Y2016 | | F | | NA | | NA | | NA | | NA | | NA |
| Takwa | Y2002/03 - Y2017 | | F | | 5.368 | | 11.735 | | 1486 | | 0.457 | | 1.000 |
| Takwa | Y2015 - Y2016 | | F | | NA | | NA | | NA | | NA | | NA |
| Takwa | Y2015 - Y2017 | | F | | 13.257 | | 11.547 | | 1486 | | 1.148 | | 1.000 |
| Takwa | Y2016 - Y2017 | | F | | NA | | NA | | NA | | NA | | NA |
| Takwa | Y2002/03 - Y2015 | | M | | 15.680 | | 7.756 | | 1486 | | 2.022 | | 0.694 |
| Takwa | Y2002/03 - Y2016 | | M | | NA | | NA | | NA | | NA | | NA |
| Takwa | Y2002/03 - Y2017 | | M | | 15.016 | | 9.116 | | 1486 | | 1.647 | | 0.756 |
| Takwa | Y2015 - Y2016 | | M | | NA | | NA | | NA | | NA | | NA |
| Takwa | Y2015 - Y2017 | | M | | -0.664 | | 7.585 | | 1486 | | -0.088 | | 1.000 |
| Takwa | Y2016 - Y2017 | | M | | NA | | NA | | NA | | NA | | NA |
| **contrast** | | **River** | | **estimate** | | **SE** | | **df** | | **p.val.adjusted** | |  |  |
| Y2002/03,F - Y2002/03,M | | Chalifour | | 40.778 | | 15.45949 | | 1486 | | 0.095 | |  |  |
| Y2015,F - Y2015,M | | Chalifour | | 15.133 | | 12.55743 | | 1486 | | 1.000 | |  |  |
| Y2016,F - Y2016,M | | Chalifour | | 42.808 | | 9.106448 | | 1486 | | 0.000 | |  |  |
| Y2017,F - Y2017,M | | Chalifour | | 49.833 | | 13.59589 | | 1486 | | 0.005 | |  |  |
| Y2002/03 F - Y2002/03 M | | Icon | | 90.397 | | 10.51359 | | 1486 | | 0.000 | |  |  |
| Y2015 F - Y2015 M | | Icon | | 42.125 | | 16.28627 | | 1486 | | 0.114 | |  |  |
| Y2016 F - Y2016 M | | Icon | | 73.531 | | 12.82151 | | 1486 | | 0.000 | |  |  |
| Y2017 F - Y2017 M | | Icon | | NA | | NA | | NA | | NA | |  |  |
| Y2002/03 F - Y2002/03 M | | Perch | | 76.993 | | 8.685832 | | 1486 | | 0.000 | |  |  |
| Y2015 F - Y2015 M | | Perch | | 27.371 | | 14.47971 | | 1486 | | 0.587 | |  |  |
| Y2016 F - Y2016 M | | Perch | | 63.822 | | 10.20524 | | 1486 | | 0.000 | |  |  |
| Y2017 F - Y2017 M | | Perch | | NA | | NA | | NA | | NA | |  |  |
| Y2002/03 F - Y2002/03 M | | Takwa | | 52.576 | | 9.310169 | | 1486 | | 0.000 | |  |  |
| Y2015 F - Y2015 M | | Takwa | | 76.146 | | 7.531934 | | 1486 | | 0.000 | |  |  |
| Y2016 F - Y2016 M | | Takwa | | NA | | NA | | NA | | NA | |  |  |
| Y2017 F - Y2017 M | | Takwa | | 62.224 | | 11.58146 | | 1486 | | 0.000 | |  |  |

Table S5 Mass lsmean with lower (LCI) and upper (UCI) 95% confidence interval

| **River** | **Year** | **Sex** | **lsmean** | **SE** | **df** | **LCI** | **UCI** |
| --- | --- | --- | --- | --- | --- | --- | --- |
| Chalifour | Y2002/03 | F | 1083.781 | 1.118 | 1481 | 871.040 | 1348.482 |
| Chalifour | Y2015 | F | 635.239 | 1.093 | 1481 | 533.144 | 756.884 |
| Chalifour | Y2016 | F | 834.081 | 1.064 | 1481 | 738.480 | 942.058 |
| Chalifour | Y2017 | F | 766.353 | 1.101 | 1481 | 634.220 | 926.015 |
| Chalifour | Y2002/03 | M | 804.122 | 1.034 | 1481 | 753.097 | 858.604 |
| Chalifour | Y2015 | M | 609.089 | 1.031 | 1481 | 573.270 | 647.146 |
| Chalifour | Y2016 | M | 632.621 | 1.030 | 1481 | 597.534 | 669.768 |
| Chalifour | Y2017 | M | 559.218 | 1.035 | 1481 | 523.025 | 597.914 |
| Icon | Y2002/03 | F | 1316.329 | 1.066 | 1481 | 1160.305 | 1493.335 |
| Icon | Y2015 | F | 677.842 | 1.125 | 1481 | 537.611 | 854.651 |
| Icon | Y2016 | F | 908.121 | 1.097 | 1481 | 757.145 | 1089.202 |
| Icon | Y2017 | F | NA | NA | 1481 | NA | NA |
| Icon | Y2002/03 | M | 778.540 | 1.052 | 1481 | 705.276 | 859.414 |
| Icon | Y2015 | M | 461.328 | 1.033 | 1481 | 432.737 | 491.807 |
| Icon | Y2016 | M | 469.495 | 1.027 | 1481 | 445.413 | 494.879 |
| Icon | Y2017 | M | 413.395 | 1.056 | 1481 | 371.688 | 459.781 |
| Perch | Y2002/03 | F | 1066.553 | 1.056 | 1481 | 957.583 | 1187.924 |
| Perch | Y2015 | F | 695.730 | 1.097 | 1481 | 580.064 | 834.460 |
| Perch | Y2016 | F | 750.420 | 1.069 | 1481 | 658.207 | 855.552 |
| Perch | Y2017 | F | NA | NA | 1481 | NA | NA |
| Perch | Y2002/03 | M | 579.106 | 1.036 | 1481 | 540.230 | 620.779 |
| Perch | Y2015 | M | 568.516 | 1.059 | 1481 | 508.060 | 636.166 |
| Perch | Y2016 | M | 438.469 | 1.039 | 1481 | 407.101 | 472.254 |
| Perch | Y2017 | M | 479.248 | 1.101 | 1481 | 396.617 | 579.095 |
| Takwa | Y2002/03 | F | 974.184 | 1.051 | 1481 | 883.485 | 1074.196 |
| Takwa | Y2015 | F | 1043.415 | 1.048 | 1481 | 951.027 | 1144.778 |
| Takwa | Y2016 | F | NA | NA | 1481 | NA | NA |
| Takwa | Y2017 | F | 959.763 | 1.078 | 1481 | 828.898 | 1111.290 |
| Takwa | Y2002/03 | M | 699.694 | 1.051 | 1481 | 635.229 | 770.702 |
| Takwa | Y2015 | M | 657.593 | 1.032 | 1481 | 618.431 | 699.234 |
| Takwa | Y2016 | M | NA | NA | 1481 | NA | NA |
| Takwa | Y2017 | M | 716.593 | 1.050 | 1481 | 651.243 | 788.501 |

Table S6 Mass contrasts. Where insufficient data existed to make a contrast, NA is indicated.

| **River** | **contrast** | | **Sex** | | **River** | | **estimate** | | **SE** | | **df** | **t.ratio** | | **p.val.adjusted** |
| --- | --- | --- | --- | --- | --- | --- | --- | --- | --- | --- | --- | --- | --- | --- |
| Chalifour | Y2002/03 - Y2015 | | F | | Chalifour | | 0.534 | | 0.143 | | 1481 | 3.741 | | 0.006 |
| Chalifour | Y2002/03 - Y2016 | | F | | Chalifour | | 0.262 | | 0.128 | | 1481 | 2.054 | | 0.214 |
| Chalifour | Y2002/03 - Y2017 | | F | | Chalifour | | 0.347 | | 0.147 | | 1481 | 2.352 | | 0.151 |
| Chalifour | Y2015 - Y2016 | | F | | Chalifour | | -0.272 | | 0.109 | | 1481 | -2.504 | | 0.151 |
| Chalifour | Y2015 - Y2017 | | F | | Chalifour | | -0.188 | | 0.131 | | 1481 | -1.427 | | 0.513 |
| Chalifour | Y2016 - Y2017 | | F | | Chalifour | | 0.085 | | 0.115 | | 1481 | 0.738 | | 1 |
| Chalifour | Y2002/03 - Y2015 | | M | | Chalifour | | 0.278 | | 0.046 | | 1481 | 6.103 | | 0 |
| Chalifour | Y2002/03 - Y2016 | | M | | Chalifour | | 0.24 | | 0.044 | | 1481 | 5.414 | | 0 |
| Chalifour | Y2002/03 - Y2017 | | M | | Chalifour | | 0.363 | | 0.048 | | 1481 | 7.606 | | 0 |
| Chalifour | Y2015 - Y2016 | | M | | Chalifour | | -0.038 | | 0.042 | | 1481 | -0.893 | | 0.952 |
| Chalifour | Y2015 - Y2017 | | M | | Chalifour | | 0.085 | | 0.046 | | 1481 | 1.856 | | 0.257 |
| Chalifour | Y2016 - Y2017 | | M | | Chalifour | | 0.123 | | 0.045 | | 1481 | 2.751 | | 0.041 |
| Icon | Y2002/03 - Y2015 | | F | | Icon | | 0.664 | | 0.135 | | 1481 | 4.933 | | 0 |
| Icon | Y2002/03 - Y2016 | | F | | Icon | | 0.371 | | 0.113 | | 1481 | 3.29 | | 0.015 |
| Icon | Y2002/03 - Y2017 | | F | | Icon | | NA | | NA | | NA | NA | | NA |
| Icon | Y2015 - Y2016 | | F | | Icon | | -0.292 | | 0.15 | | 1481 | -1.947 | | 0.315 |
| Icon | Y2015 - Y2017 | | F | | Icon | | NA | | NA | | NA | NA | | NA |
| Icon | Y2016 - Y2017 | | F | | Icon | | NA | | NA | | NA | NA | | NA |
| Icon | Y2002/03 - Y2015 | | M | | Icon | | 0.523 | | 0.06 | | 1481 | 8.719 | | 0 |
| Icon | Y2002/03 - Y2016 | | M | | Icon | | 0.506 | | 0.057 | | 1481 | 8.859 | | 0 |
| Icon | Y2002/03 - Y2017 | | M | | Icon | | 0.633 | | 0.074 | | 1481 | 8.553 | | 0 |
| Icon | Y2015 - Y2016 | | M | | Icon | | -0.018 | | 0.042 | | 1481 | -0.415 | | 1 |
| Icon | Y2015 - Y2017 | | M | | Icon | | 0.11 | | 0.063 | | 1481 | 1.734 | | 0.315 |
| Icon | Y2016 - Y2017 | | M | | Icon | | 0.127 | | 0.06 | | 1481 | 2.103 | | 0.201 |
| Perch | Y2002/03 - Y2015 | | F | | Perch | | 0.427 | | 0.108 | | 1481 | 3.965 | | 0.001 |
| Perch | Y2002/03 - Y2016 | | F | | Perch | | 0.352 | | 0.087 | | 1481 | 4.063 | | 0.001 |
| Perch | Y2002/03 - Y2017 | | F | | Perch | | NA | | NA | | NA | NA | | NA |
| Perch | Y2015 - Y2016 | | F | | Perch | | -0.076 | | 0.114 | | 1481 | -0.662 | | 1 |
| Perch | Y2015 - Y2017 | | F | | Perch | | NA | | NA | | NA | NA | | NA |
| Perch | Y2016 - Y2017 | | F | | Perch | | NA | | NA | | NA | NA | | NA |
| Perch | Y2002/03 - Y2015 | | M | | Perch | | 0.018 | | 0.067 | | 1481 | 0.274 | | 1 |
| Perch | Y2002/03 - Y2016 | | M | | Perch | | 0.278 | | 0.052 | | 1481 | 5.367 | | 0 |
| Perch | Y2002/03 - Y2017 | | M | | Perch | | 0.189 | | 0.103 | | 1481 | 1.842 | | 0.382 |
| Perch | Y2015 - Y2016 | | M | | Perch | | 0.26 | | 0.069 | | 1481 | 3.782 | | 0.003 |
| Perch | Y2015 - Y2017 | | M | | Perch | | 0.171 | | 0.112 | | 1481 | 1.522 | | 0.513 |
| Perch | Y2016 - Y2017 | | M | | Perch | | -0.089 | | 0.104 | | 1481 | -0.858 | | 1 |
| Takwa | Y2002/03 - Y2015 | | F | | Takwa | | -0.069 | | 0.069 | | 1481 | -1 | | 1 |
| Takwa | Y2002/03 - Y2016 | | F | | Takwa | | NA | | NA | | NA | NA | | NA |
| Takwa | Y2002/03 - Y2017 | | F | | Takwa | | 0.015 | | 0.09 | | 1481 | 0.166 | | 1 |
| Takwa | Y2015 - Y2016 | | F | | Takwa | | NA | | NA | | NA | NA | | NA |
| Takwa | Y2015 - Y2017 | | F | | Takwa | | 0.084 | | 0.088 | | 1481 | 0.945 | | 1 |
| Takwa | Y2016 - Y2017 | | F | | Takwa | | NA | | NA | | NA | NA | | NA |
| Takwa | Y2002/03 - Y2015 | | M | | Takwa | | 0.062 | | 0.058 | | 1481 | 1.063 | | 1 |
| Takwa | Y2002/03 - Y2016 | | M | | Takwa | | NA | | NA | | NA | NA | | NA |
| Takwa | Y2002/03 - Y2017 | | M | | Takwa | | -0.024 | | 0.069 | | 1481 | -0.344 | | 1 |
| Takwa | Y2015 - Y2016 | | M | | Takwa | | NA | | NA | | NA | NA | | NA |
| Takwa | Y2015 - Y2017 | | M | | Takwa | | -0.086 | | 0.058 | | 1481 | -1.483 | | 1 |
| Takwa | Y2016 - Y2017 | | M | | Takwa | | NA | | NA | | NA | NA | | NA |
| **contrast** | | **River** | | **estimate** | | **SE** | | **df** | | **p.val.adjusted** | | |  |  |
| Y2002/03,F - Y2002/03,M | | Chalifour | | 0.298 | | 0.116 | | 1481 | | 0.130 | | |  |  |
| Y2015,F - Y2015,M | | Chalifour | | 0.042 | | 0.095 | | 1481 | | 1.000 | | |  |  |
| Y2016,F - Y2016,M | | Chalifour | | 0.276 | | 0.069 | | 1481 | | 0.002 | | |  |  |
| Y2017,F - Y2017,M | | Chalifour | | 0.315 | | 0.102 | | 1481 | | 0.034 | | |  |  |
| Y2002/03 F - Y2002/03 M | | Icon | | 0.525 | | 0.082 | | 1481 | | 0.000 | | |  |  |
| Y2015 F - Y2015 M | | Icon | | 0.385 | | 0.123 | | 1481 | | 0.025 | | |  |  |
| Y2016 F - Y2016 M | | Icon | | 0.660 | | 0.097 | | 1481 | | 0.000 | | |  |  |
| Y2017 F - Y2017 M | | Icon | | NA | | NA | | NA | | NA | | |  |  |
| Y2002/03 F - Y2002/03 M | | Perch | | 0.611 | | 0.065 | | 1481 | | 0.000 | | |  |  |
| Y2015 F - Y2015 M | | Perch | | 0.202 | | 0.109 | | 1481 | | 0.577 | | |  |  |
| Y2016 F - Y2016 M | | Perch | | 0.537 | | 0.077 | | 1481 | | 0.000 | | |  |  |
| Y2017 F - Y2017 M | | Perch | | NA | | NA | | NA | | NA | | |  |  |
| Y2002/03 F - Y2002/03 M | | Takwa | | 0.331 | | 0.070 | | 1481 | | 0.000 | | |  |  |
| Y2015 F - Y2015 M | | Takwa | | 0.462 | | 0.057 | | 1481 | | 0.000 | | |  |  |
| Y2016 F - Y2016 M | | Takwa | | NA | | NA | | NA | | NA | | |  |  |
| Y2017 F - Y2017 M | | Takwa | | 0.292 | | 0.089 | | 1481 | | 0.025 | | |  |  |

Sex Bias


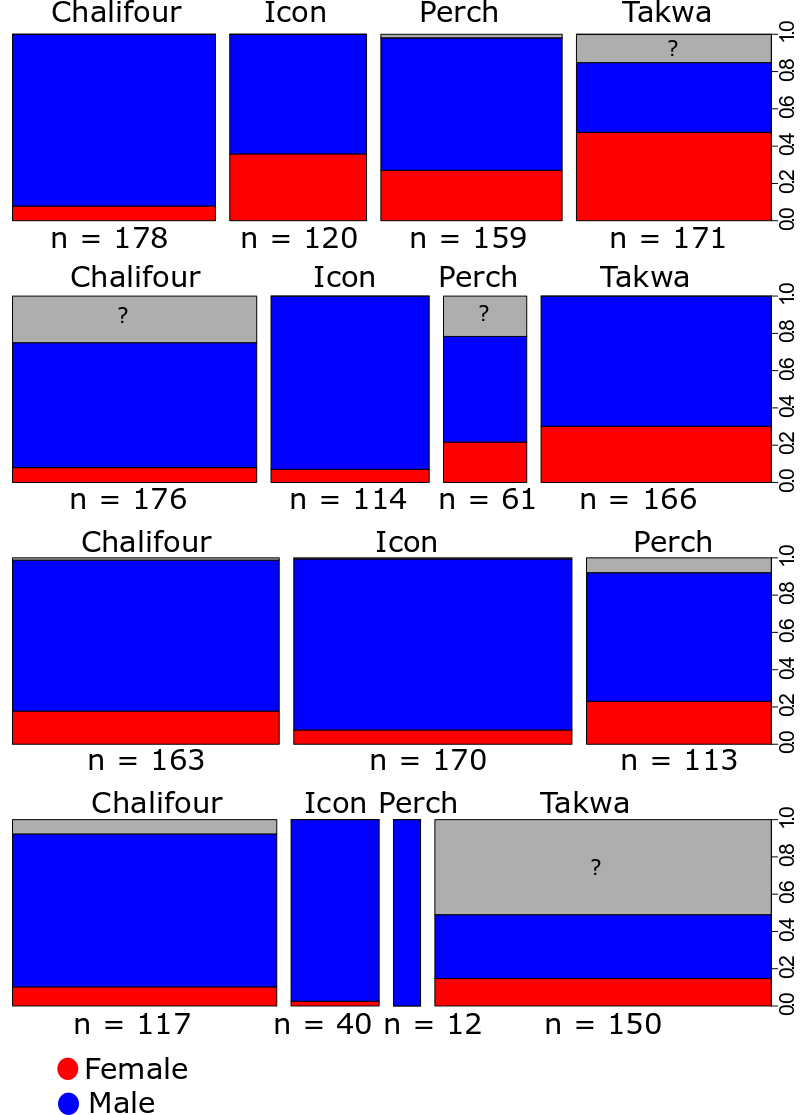


2002/03

2015

2016

2017

Figure S1 Sex-bias at sampling sites, where blue = males, red = females and grey = unknowns in 2002-03, 2015, 2016 and 2017. Also shown are the total captured walleye in each river (n).

*Genomics*

Table S7 Number of individuals successfully sequenced and retained after all filtering (n), number of private alleles (private), number of SNPs (SNPs), percent polymorphic loci (% poly), expected heterozygosity (HE), observed heterozygosity (HO), pi (π).

| **Population & year** | **n** | **Private** | **SNPs** | **% poly** | **H_E_** | **StdErr** | **H_O_** | **StdErr** | **π** | **StdErr** |
| --- | --- | --- | --- | --- | --- | --- | --- | --- | --- | --- |
| **Chalifour 2003** | 27.5725 | 12 | 7995 | 0.1792 | 0.2302 | 0.0019 | 0.2256 | 0.002 | 0.2345 | 0.0019 |
| **Chalifour 2015** | 46.8723 | 14 | 9236 | 0.1961 | 0.2264 | 0.0018 | 0.2251 | 0.0019 | 0.2289 | 0.0018 |
| **Icon-Perch 2003** | 83.9165 | 53 | 7028 | 0.1838 | 0.2134 | 0.002 | 0.2014 | 0.0019 | 0.2146 | 0.002 |
| **Icon-Perch 2015** | 85.3662 | 44 | 9196 | 0.2017 | 0.2183 | 0.0018 | 0.2099 | 0.0018 | 0.2196 | 0.0018 |
| **Takwa 2003** | 33.3336 | 51 | 9253 | 0.1868 | 0.2223 | 0.0018 | 0.2141 | 0.0018 | 0.2257 | 0.0018 |
| **Takwa 2015** | 35.9956 | 7 | 8031 | 0.1754 | 0.2113 | 0.0019 | 0.2011 | 0.0019 | 0.2143 | 0.002 |

Table S8 Outlier loci, PC axis on which they are relevant, and explanation of the population structure explained by each PC axis for historic-contemporary contrasts for each population separately and all populations combined. Figures S2 to S16 illustrate the results described in this table.

| **Population** | **Num. of individuals in each analysis: all (unknowns plus m & f) and for each sex separately** | **Num. of SNPs used in contrast** | **Num. of outliers** | **% of outliers (NS for no population structure)** | **Population structure explained by each PC when outliers are included in the data** | **Num. of outlier loci associated with relevant PC/total no. of outliers** |
| --- | --- | --- | --- | --- | --- | --- |
| **All populations and years, global** | all, 342 | 8728 | 70 | 0.80 | PC1 separates north from the south, and also Pe03 and Ch03/15 from Ic03/15/Pe15 marginally; PC2 separates Ch03/15 from the other populations, and also Pe03 from the other individuals. Outliers do not maintain the population structure. | PC1, 55/70; PC2, 15/70 |
|  | f, 118 | 6613 | 11 | 0.17 | PC1 separates north from the south, and also Pe03 and Ch03/15 from Ic03/15/Pe15 marginally; PC2 separates Ch03 from Ch15, and both Chalifour years from the other southern populations. Outliers do not maintain the population structure. | PC1, 9/11; PC2, 2/11 |
|  | m, 213 | 9958 | 151 | 1.52 | PC1 separates north from the south, and also Pe03 and Ch03/15 from Ic03/15/Pe15 marginally; PC2 separates Ch03/15 from the other populations, Ch03 from Ch15, and also Pe03 from the other individuals. Outliers maintain population structure in Ch. | PC1, 91/151; PC2, 60/151 |
| **south** | all, 268 | 7358 | 51 | 0.69 | PCI separates Ch from other pops, PC2 separates Ch 03 from 15 and Pe03. Outliers maintain population structure in Ch. | PC1, 15/51; PC2, 36/51 |
|  | f, 83 | 6547 | 65 | 0.99 | PC1 separates Ch03/15 from the other sites. PC2 separates Pe03 and Cha03 from the other sites. Outliers maintain population structure. | PC1, 17/65; PC2, 48/65 |
|  | m, 176 | 8068 | 69 | 0.86 | PC1 separates Ch03/15 from other sites. PC2 separates Chali03 from 2015 and Pe03 a bit from rest of that group. Outliers maintain population structure | PC1, 18/69; PC2, 51/69 |
| **Chalifour** | all, 79 | 8513 | 129 | 1.52 | PC1, separates years. Outliers maintain population structure. | PC1, 109/129; PC2, 20/129 |
|  | f, 17 | 3665 | 25 | 0.68 | PC1, separates years. Outliers do not maintain population structure | PC1, 16/25; PC2, 9/25 |
|  | m, 61 | 9294 | 263 | 2.83 | PC1, separates years. Outliers maintain population structure. | PC1, 233/263; PC2, 30/263 |
| **Icon-Perch metapopulation** | all, 189 | 6802 | 33 | 0.49 | PC2 separates Pe03 from the other populations. Outliers maintain population structure | PC2, 8/33 |
|  | f, 66 | 6442 | 73 | 1.13 | PC1 separates Pe03 from the other populations. Outliers maintain population structure. | PC1, 63/73 outliers |
|  | m, 115 | 6452 | NS | NS | NS | NS |
| **Takwa** | all, 74 | 7820 | NS | NS | NS | NS |
|  | f, 35 | 6543 | NS | NS | NS | NS |
|  | m, 37 | 10540 | NS | NS | NS | NS |
| **^ loci were not required to be present in all populations to be used (filter requirements were the same as for the main dataset** | | | | | | |

Table S9 Outlier loci blasted, mapped and annotated using blast2go. Annotation for gene ontology level 2. Biological process (BP), Molecular function (MF) and Cellular component (CC).

| **Popul-ation** | **Sex** | **Number of sequences blasted** | **Number of alleles mapped** | **Number of alleles annotated** | **Annotation (Gene Ontology = 2)** | | |
| --- | --- | --- | --- | --- | --- | --- | --- |
|  |  |  |  |  | **BP** | **MF** | **CC** |
| Cha | all | 253 | 3 | 2 | Localization | binding | cell part, organelle part, organelle, cell, membrane, protein containing complex |
| Cha | f | 46 | 0 | 4 | biological regulation, signaling, response to stimulus, regulation of biological process, cellular process, metabolic process, localization, negative regulation of biological process, positive regulation of biological process, cellular component organisation or biogenesis, multicellular organismal process | binding, transporter activity, catalytic activity | organelle, cell, cell part, synapse, cell junction, protein-containing complex, synapse part, membrane-enclosed lumen, organelle part, membrane part, membrane |
| Cha | m | 513 | 7 | 6 | biological regulation, signaling, localization, response to stimulus, regulation of biological process, negative regulation of biological process, cellular component organisation of biogenesis, developmental process, multicellular organismal process, cellular process, cell aggregation, biological adhesion, behavior, metabolic process, positive regulation of biological process, growth, multi-organismal process | binding, molecular transducer activity, structural molecule activity, transporter activity | cell, protein-containing complex, cell part, organelle, organelle part, membrane, synapse, supermolecular complex, cell junction, synapse part, extracellular region, extracellular region part, membrane part |
| IcPe | all | 64 | 0 | 2 | negative regulation of biological process, biological regulation, metabolic process, signaling, response to stimulus, cellular process, regulation of biological process | catalytic activity, binding | cell, cell part |
| IcPe | f | 144 | 0 | 2 | signaling, response to stimulus, regulation of biological process, growth, locomotion, developmental process, multicellular organismal process, cellular process, biological regulation, metabolic process, localization, reproductive process, negative regulation of biological process, positive regulation of biological process, cell population proliferation, multi-organism process, cellular component organization or biogenesis, reproduction | molecular transducer activity, transcription regulator activity, binding | organelle, cell, membrane-enclosed lumen, cell part, organelle part, protein-containing complex |
| Sou | all | 128 | 0 | 2 | signaling, response to stimulus, regulation of biological process, growth, locomotion, developmental process, multicellular organismal process, cellular process, biological regulation, metabolic process, localization, reproductive process, negative regulation of biological process, positive regulation of biological process, cell population proliferation, multi-organism process, cellular component organization of biogenesis, reproduction | molecular transducer activity, transcription regulator activity, binding | organelle, cell, membrane-enclosed lumen, cell part, organelle part, protein-containing complex |

Outlier analysis results presentation

For each configuration of populations assessed, results for m and f combined are followed by results for each sex separately. Meaningful population structure that allowed for outlier detection was present in most cases, sometimes based on geography and sometimes temporal between timepoints.


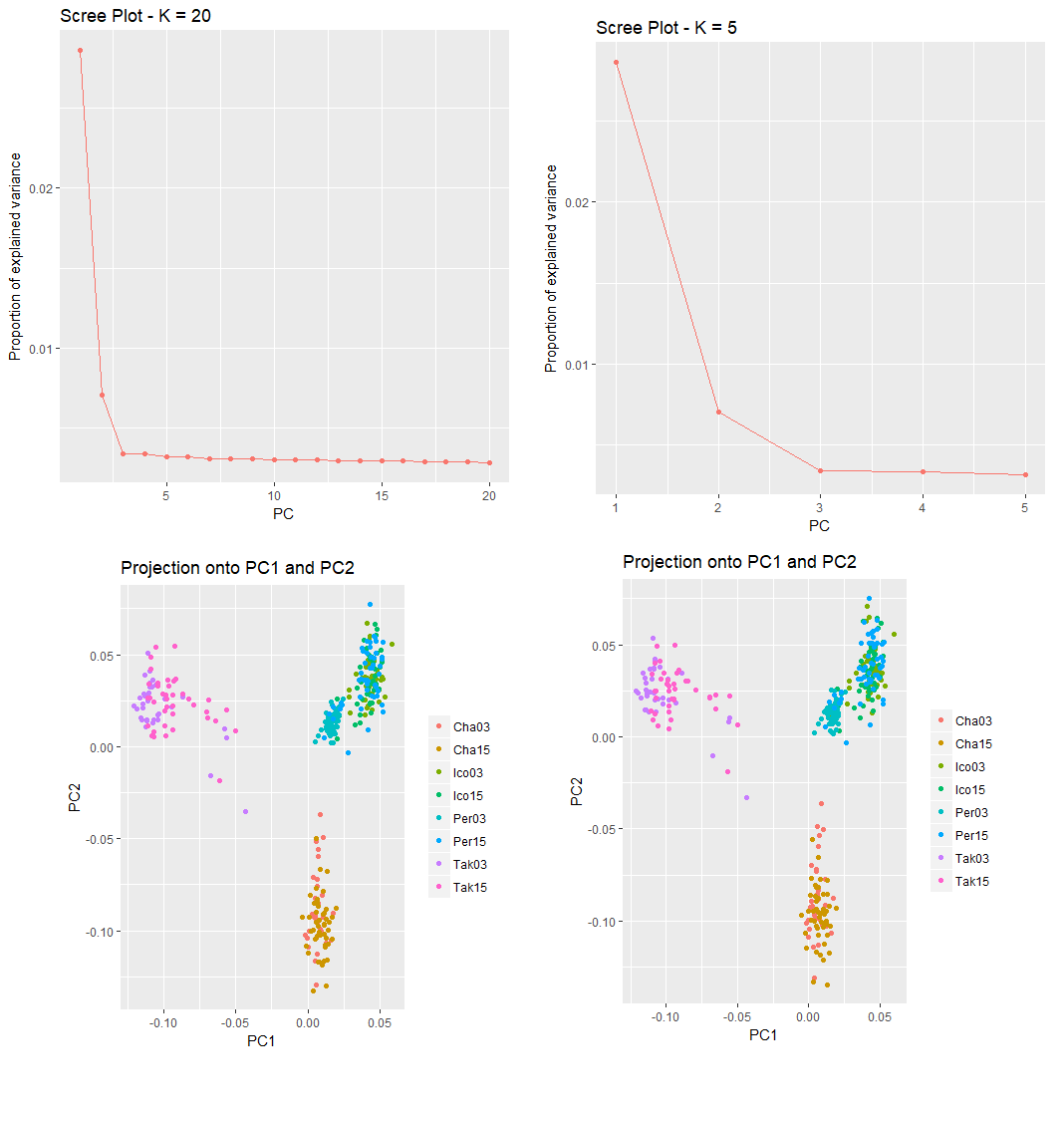


Figure S2 All populations, all years, m and f combined, k=2. Top: scree panels for selecting best k. Bottom left, with outliers left in. Bottom right, excluding outliers. Result: PC1 separates the north from the south, and also Icon03/15Perch15 a bit, and the second PC separates Chalifour from the other pops. Shown in the SNP list that associates SNPs with PCs below, the vast majority of outliers are associated with PC1 (15 to PC2). However, removing outliers does not change the structure in a meaningful way.

##overall outliers

> print(ID[outliers])

[1] "2764_47" "5777_26" "7221_52" "7315_29" "9664_69" "10060_79" "10881_53"

[8] "11507_79" "12647_55" "14845_11" "15362_57" "15426_49" "16571_28" "23892_66"

[15] "25728_47" "26281_46" "27021_71" "28192_35" "29788_76" "31425_7" "31886_71"

[22] "34289_47" "35235_60" "36864_48" "37605_43" "38039_35" "39685_62" "41527_40"

[29] "41635_58" "45518_67" "45711_42" "46331_65" "48658_18" "49504_49" "50030_36"

[36] "50404_26" "51264_20" "53498_56" "62465_26" "65438_60" "65770_56" "69567_54"

[43] "69971_62" "71554_26" "72916_70" "74122_76" "74452_59" "75343_34" "76517_75"

[50] "81466_14" "83593_61" "84174_76" "85630_70" "88020_72" "88250_71" "89842_9"

[57] "91022_14" "91315_70" "91507_23" "93560_78" "93677_42" "97401_34" "97840_17"

[64] "99863_28" "99888_60" "101083_6" "102928_19" "103420_43" "104231_8" "104441_34"

=70 outlier loci --- note that did not require all loci to be present in all pops here, so can’t really just divide by total number of SNPs to get the percent outliers. Average number of SNPs per pop was 7817, but ranged between 3766 and 8694.

> snp_pc <- get.pc(x, outliers)

> snp_pc

SNP PC

1 99 1

2 341 1

3 456 1

4 468 2

5 671 2

6 708 1

7 779 1

8 832 1

9 944 1

10 1151 1

11 1190 1

12 1199 1

13 1292 2

14 1938 1

15 2122 1

16 2172 2

17 2254 2

18 2347 1

19 2487 1

20 2639 1

21 2684 1

22 2914 1

23 3010 1

24 3154 1

25 3227 1

26 3269 1

27 3408 1

28 3556 1

29 3562 1

30 3882 2

31 3900 1

32 3956 1

33 4139 1

34 4204 1

35 4241 2

36 4265 1

37 4315 2

38 4444 2

39 4871 1

40 5035 1

41 5063 1

42 5301 1

43 5320 1

44 5464 1

45 5583 2

46 5699 1

47 5733 1

48 5813 2

49 5907 1

50 6398 2

51 6602 1

52 6662 1

53 6781 2

54 6974 1

55 7000 1

56 7139 1

57 7244 1

58 7269 1

59 7287 1

60 7498 1

61 7515 1

62 7867 1

63 7896 1

64 8078 2

65 8081 1

66 8190 1

67 8356 1

68 8405 2

69 8489 1

70 8511 1


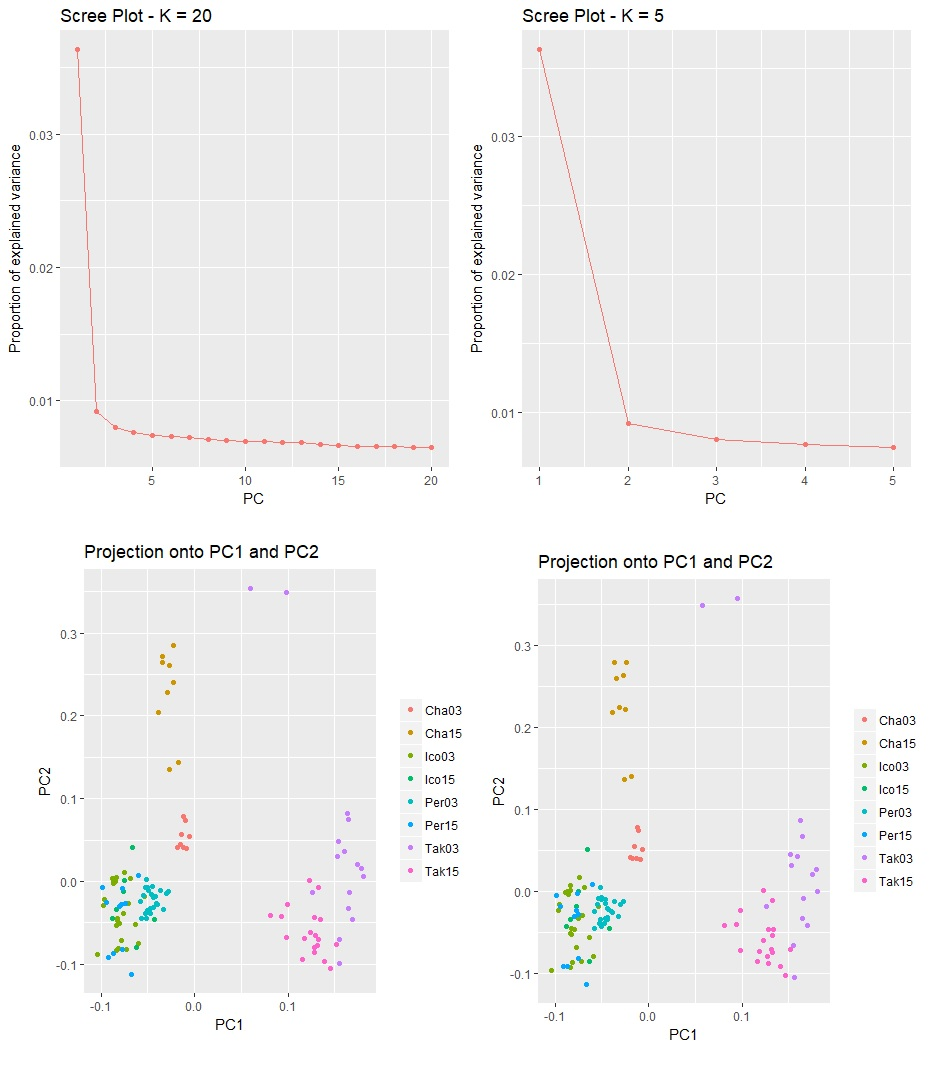


Figure S3 All populations and years, f, k=2. Top panels are scree plots for determining the best k. Bottom panels are: (left) including outliers, and (right) excluding outliers. Result: PC1 separates north and south, and maybe Chali03/15 and Pe03 a little bit from the Ic03,15,Pe15 group. PC2 separates Ch03 from Ch15, and also both Chalifour years from the other southern pops. Result looks similar to when M and F are combined, except that PC2 also separates Ch03 and 15. Removing outliers has no meaningful affect on the population structure. number of pops that I required loci to be present in for the full dataset is 6/8.

> print(ID[outliers])

[1] "2764_47" "12315_60" "13844_69" "16571_28" "27138_61" "34289_47" "36864_48"

[8] "45711_42" "89842_9" "93560_78" "103420_43"

=11/6613 SNPs, but not all SNPs would have been present in all pops

> snp_pc

SNP PC

1 70 1

2 669 2

3 774 2

4 950 1

5 1696 1

6 2224 1

7 2410 1

8 2970 1

9 5472 1

10 5739 1

11 6434 1


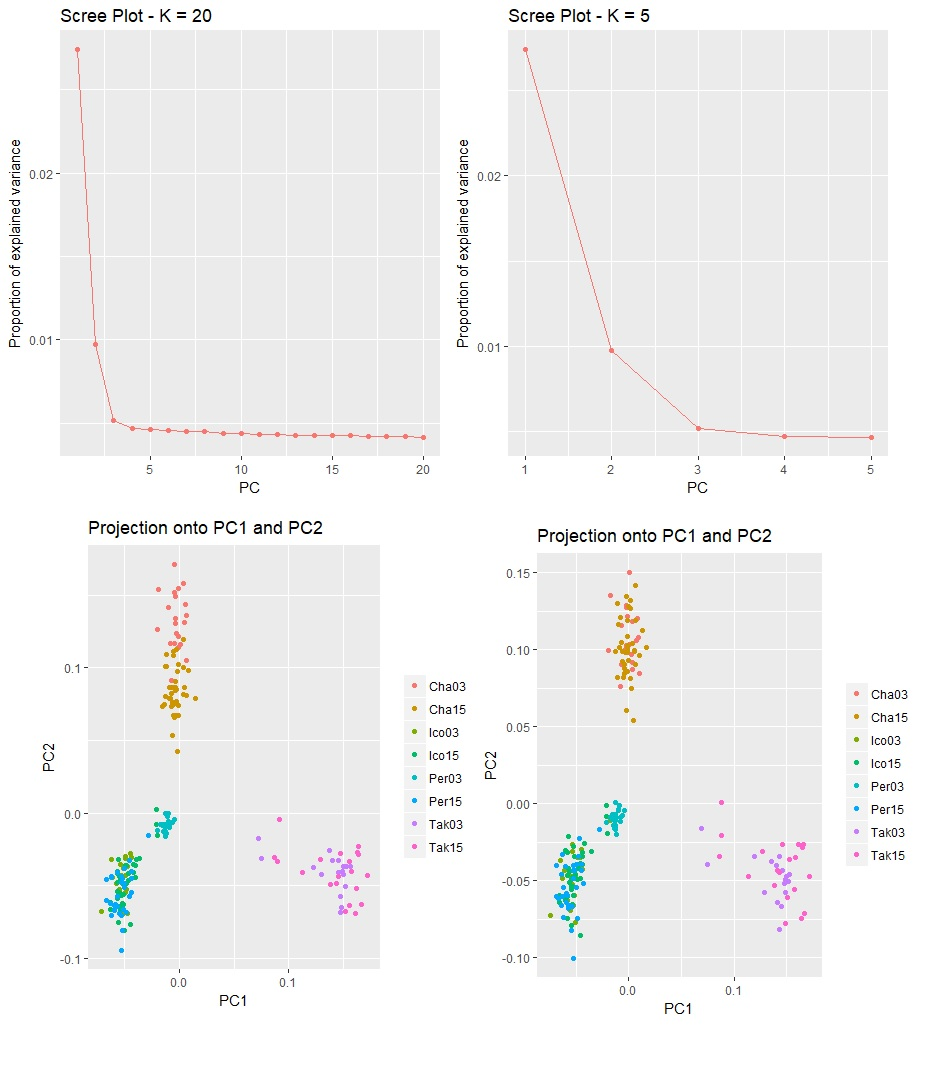


Figure S4 All populations and years, m, k=2. Top panels are scree plots used to determine best k. Bottom panels are: (left) including outliers, and (right) excluding outliers. Result: PC1 separates north from south, and also Pe03 and the Chalifours. PC2 separates Cha03 and 15 from the other pops, Pe03 from the other pops, and Ch03 from 15. Removing outliers removes the structure between Ch03 and 15.

> print(ID[outliers])

[1] "1721_78" "2468_66" "2764_47" "2916_78" "5486_58" "5777_26" "7221_52"

[8] "7232_14" "7393_79" "8976_18" "9415_78" "9629_79" "9664_69" "9942_79"

[15] "10060_79" "10157_79" "10696_78" "10881_53" "11507_79" "12647_55" "12994_48"

[22] "14431_76" "15362_57" "15426_49" "16571_28" "18456_6" "19262_77" "19850_46"

[29] "21844_79" "22374_17" "23892_66" "23960_64" "24841_61" "25728_47" "26281_46"

[36] "26667_79" "27021_71" "28192_35" "29164_79" "29788_76" "31135_79" "31385_26"

[43] "31886_71" "32228_78" "33113_74" "33150_19" "33608_78" "34382_79" "34803_77"

[50] "34842_79" "35235_60" "35988_62" "36864_48" "37605_43" "38039_35" "38403_14"

[57] "38443_39" "39301_13" "39685_62" "41527_40" "41635_58" "41986_78" "42743_77"

[64] "43077_77" "43351_78" "44213_21" "44786_79" "45557_77" "45606_14" "45711_42"

[71] "46331_65" "47350_50" "48658_18" "49504_49" "50030_36" "50404_26" "51264_20"

[78] "53093_25" "53498_56" "54744_32" "58417_15" "59518_78" "61672_14" "62465_26"

[85] "62534_37" "62632_56" "63988_78" "65438_60" "65770_56" "66402_17" "67781_28"

[92] "69537_35" "69567_54" "71145_48" "71554_26" "71655_77" "72447_18" "72944_78"

[99] "76517_75" "77651_61" "77767_20" "78676_53" "79047_76" "79305_23" "79365_74"

[106] "80664_78" "81466_14" "82281_25" "82349_79" "82679_76" "83593_61" "83684_34"

[113] "84174_76" "84455_39" "85630_70" "86067_79" "87113_79" "87783_79" "87972_79"

[120] "88020_72" "88250_71" "88935_12" "89842_9" "90459_77" "91022_14" "91315_70"

[127] "91507_23" "91852_78" "93560_78" "93677_42" "94056_12" "94159_79" "94696_71"

[134] "95326_42" "96032_32" "96940_76" "97401_34" "97840_17" "99888_60" "101049_10"

[141] "101083_6" "101770_56" "102928_19" "103420_43" "103612_10" "104231_8" "104441_34"

[148] "113216_68" "114365_30" "117698_63" "123135_44"

=151 outliers

#interesting that there are so many more outliers for males

#variable number of SNPs per population

> snp_pc

SNP PC

1 31 2

2 95 1

3 118 1

4 135 2

5 366 1

6 401 1

7 537 1

8 539 1

9 560 2

10 695 2

11 743 2

12 765 2

13 769 2

14 803 2

15 813 1

16 824 2

17 878 2

18 896 1

19 958 1

20 1089 1

21 1123 1

22 1283 2

23 1381 1

24 1391 2

25 1498 2

26 1679 1

27 1759 2

28 1831 1

29 2030 2

30 2090 2

31 2241 1

32 2251 2

33 2343 1

34 2434 1

35 2493 2

36 2548 2

37 2582 2

38 2685 1

39 2782 2

40 2842 1

41 2980 2

42 3005 1

43 3061 1

44 3096 2

45 3187 1

46 3192 1

47 3245 2

48 3325 2

49 3373 2

50 3377 2

51 3424 1

52 3507 1

53 3580 1

54 3661 1

55 3708 1

56 3736 1

57 3739 1

58 3824 1

59 3862 1

60 4028 1

61 4035 1

62 4067 2

63 4140 1

64 4171 2

65 4201 2

66 4279 1

67 4328 2

68 4410 2

69 4413 1

70 4423 1

71 4486 1

72 4580 1

73 4686 2

74 4754 1

75 4800 2

76 4829 1

77 4893 2

78 5027 1

79 5054 2

80 5147 2

81 5357 1

82 5400 2

83 5511 1

84 5557 1

85 5563 1

86 5569 1

87 5656 2

88 5750 1

89 5782 1

90 5836 2

91 5926 1

92 6059 1

93 6061 1

94 6198 1

95 6245 1

96 6252 2

97 6332 2

98 6384 2

99 6747 1

100 6876 1

101 6885 1

102 6968 1

103 7009 2

104 7037 1

105 7047 1

106 7189 2

107 7286 2

108 7374 1

109 7387 2

110 7427 2

111 7510 1

112 7524 1

113 7574 1

114 7598 1

115 7705 2

116 7748 2

117 7841 2

118 7907 2

119 7927 2

120 7931 1

121 7954 1

122 8030 1

123 8114 1

124 8179 2

125 8228 1

126 8256 1

127 8276 1

128 8315 2

129 8512 1

130 8528 1

131 8576 1

132 8588 2

133 8647 1

134 8712 1

135 8782 1

136 8871 2

137 8924 1

138 8960 1

139 9159 1

140 9275 1

141 9281 1

142 9340 1

143 9460 2

144 9513 2

145 9543 1

146 9612 1

147 9636 2

148 9784 1

149 9804 1

150 9866 1

151 9914 1

#60 outliers are associated with PC2


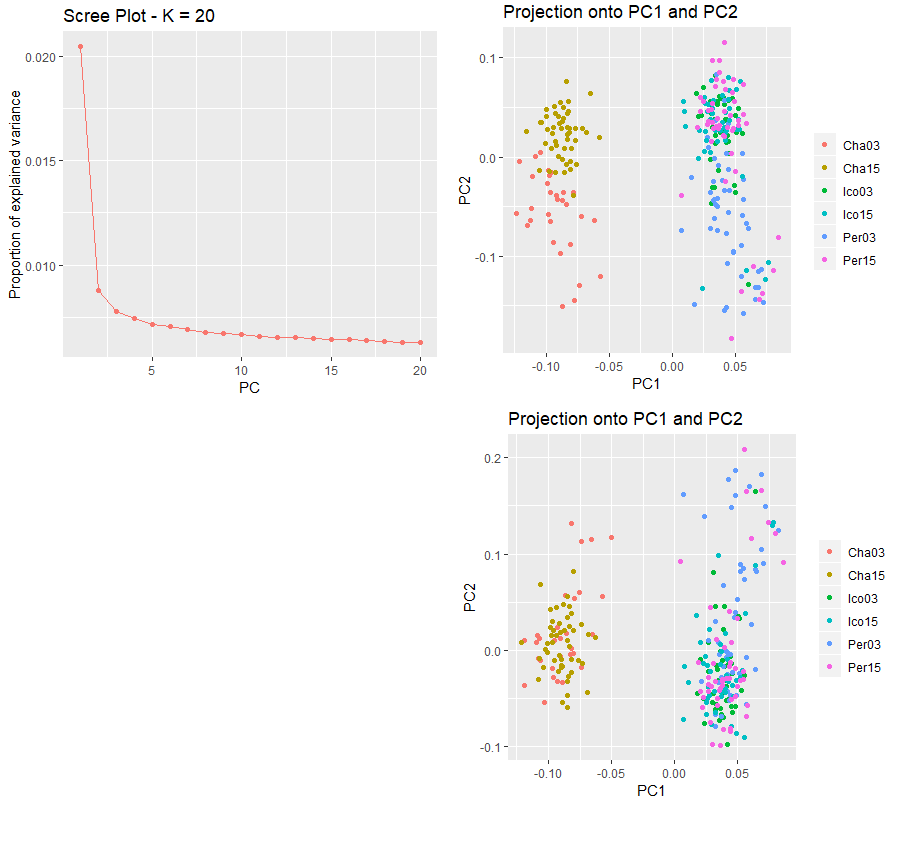


Figure S5 Southern rivers, m & f combined, k=2. Top right panel is with outliers included and bottom right panel is outliers excluded. Conclusions: structure is sufficient to detect outliers. PCI separates Ch from other pops, PC2 separates Ch 03 from 15 and Pe03. Removing outliers removes the structure.

[1] "866_78" "3193_79" "4975_78" "5302_79" "7583_78" "8206_78" "9415_78" "9942_79"

[9] "10957_77" "11235_78" "16571_28" "23360_77" "24957_79" "27021_71" "28075_78" "29208_61"

[17] "29693_78" "31135_79" "34289_47" "34803_77" "34842_79" "35262_76" "36253_79" "37605_43"

[25] "42691_45" "47695_78" "48658_18" "49486_6" "53498_56" "56508_40" "62534_37" "70726_79"

[33] "72944_78" "74122_76" "74436_48" "74568_79" "75276_78" "78450_6" "83973_79" "84111_22"

[41] "87113_79" "88895_78" "89820_78" "92110_78" "96076_79" "99202_76" "101300_12" "102745_64"

[49] "103420_43" "104310_77" "104441_34"

> snp_pc

SNP PC

1 10 2

2 127 2

3 231 2

4 250 2

5 413 2

6 457 2

7 542 2

8 585 2

9 665 2

10 684 2

11 1110 1

12 1617 2

13 1747 2

14 1928 1

15 1997 2

16 2076 2

17 2118 2

18 2242 2

19 2503 1

20 2552 1

21 2556 2

22 2596 2

23 2682 2

24 2782 1

25 3137 2

26 3486 2

27 3550 1

28 3612 2

29 3815 1

30 3948 1

31 4172 1

32 4584 2

33 4774 2

34 4865 1

35 4894 1

36 4902 2

37 4957 2

38 5210 2

39 5687 2

40 5694 2

41 5902 2

42 6051 2

43 6119 2

44 6305 1

45 6664 2

46 6896 2

47 7053 1

48 7159 2

49 7217 1

50 7296 2

51 7308 1

Pc1, 15/51


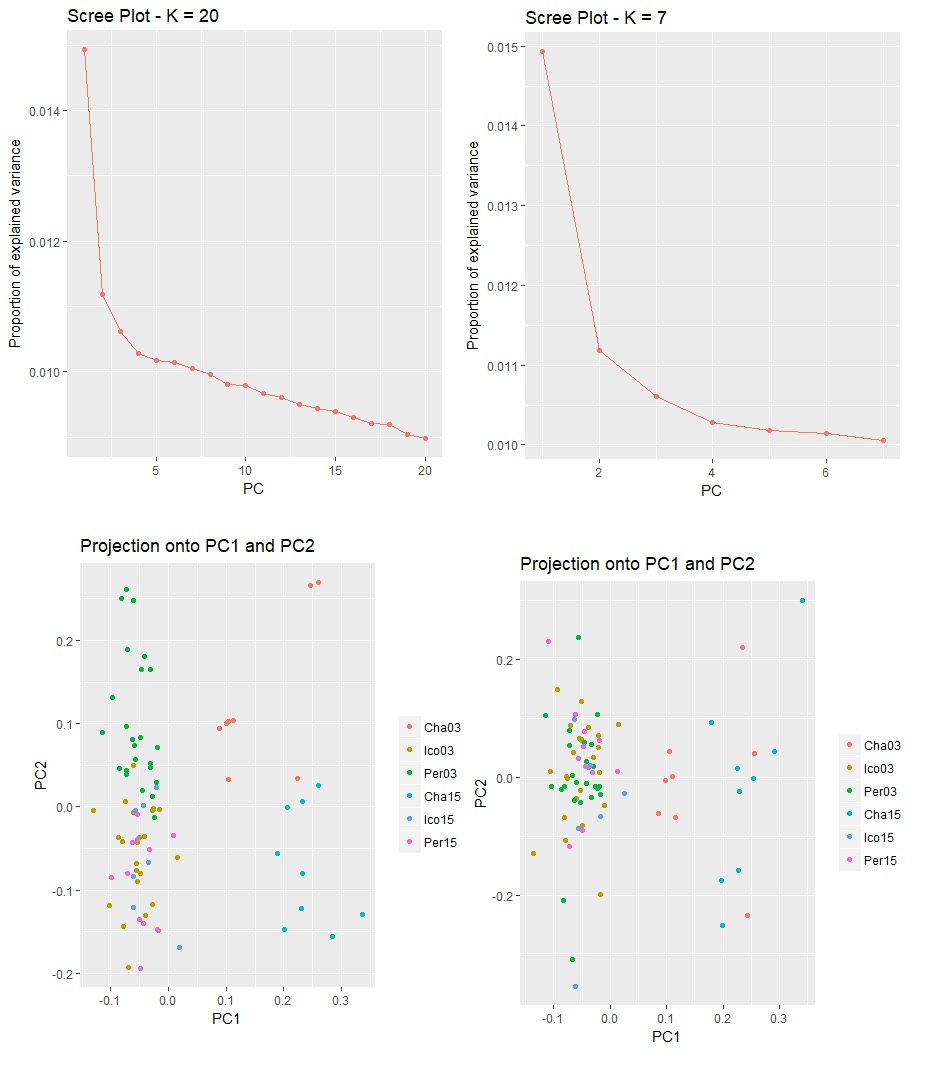


Figure S6 southern rivers, f, k=2. Top panels are the scree plots used to find the best k. Bottom panels are: (left) including outliers and (right) excluding outliers. Result: PC1 separates Chalifour from the other sites and PC2 separates Pe03 and Cha03 from the other sites. Removing outliers collapses PC2 structure, collapsing Pe03 into the other southern rivers, and Ch03 and 15. This result is the inverse of the result when both sexes are analyzed together.

> print(ID[outliers])

[1] "2003_6" "2179_39" "3193_79" "4975_78" "6298_8" "7025_29" "7583_78"

[8] "8206_79" "9533_59" "10957_77" "11815_79" "16571_28" "17580_8" "18297_78"

[15] "20589_78" "22042_79" "23360_77" "23519_75" "24957_79" "25319_5" "27138_61"

[22] "28075_78" "28650_42" "29693_78" "31509_74" "32700_77" "32789_78" "33821_6"

[29] "34289_47" "34803_77" "34842_79" "35262_76" "35866_16" "41258_78" "46439_76"

[36] "46573_78" "46592_79" "47695_78" "48338_67" "48757_78" "50171_79" "53917_60"

[43] "55665_78" "57534_66" "62150_77" "70726_79" "71278_78" "74216_22" "74568_79"

[50] "75276_78" "77273_78" "79332_79" "80680_79" "83973_79" "88895_78" "91894_55"

[57] "92110_78" "94277_78" "95663_78" "96076_79" "96628_71" "99202_76" "99476_17"

[64] "102675_77" "104310_77"

=65/6547 SNPs = 0.99% of loci are outliers

> snp_pc

SNP PC

1 37 1

2 47 1

3 110 2

4 200 2

5 271 1

6 308 1

7 346 2

8 385 2

9 464 1

10 555 2

11 611 2

12 938 1

13 995 1

14 1047 2

15 1206 2

16 1310 2

17 1398 2

18 1411 2

19 1524 2

20 1547 2

21 1694 1

22 1749 2

23 1787 1

24 1856 2

25 2002 2

26 2098 2

27 2106 1

28 2190 1

29 2217 1

30 2263 1

31 2266 2

32 2298 2

33 2337 2

34 2679 2

35 2987 2

36 2989 2

37 2990 2

38 3070 2

39 3107 1

40 3133 2

41 3223 2

42 3383 1

43 3449 2

44 3515 1

45 3663 2

46 4036 2

47 4086 2

48 4305 1

49 4329 2

50 4369 2

51 4511 2

52 4662 2

53 4765 2

54 5024 2

55 5361 2

56 5573 2

57 5595 2

58 5782 2

59 5892 2

60 5923 2

61 5959 2

62 6137 2

63 6154 2

64 6367 2

65 6495 2


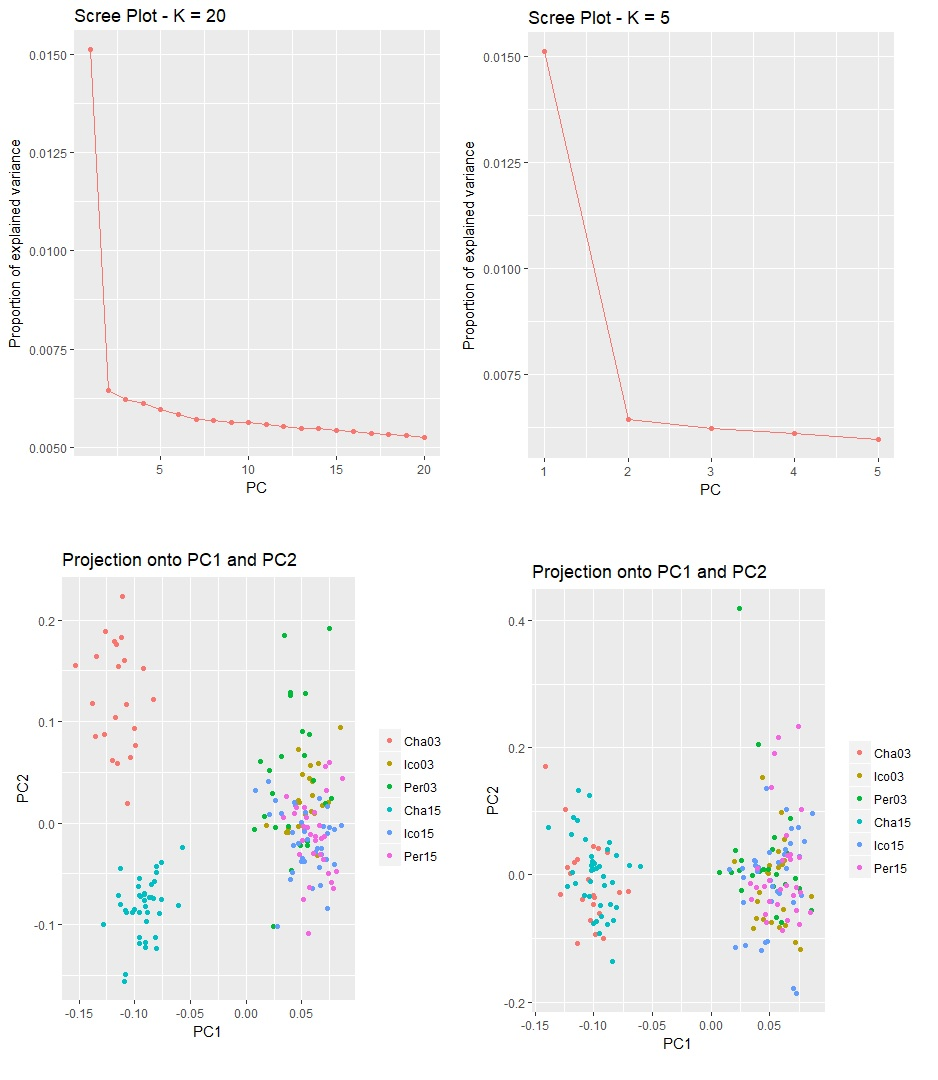


Figure S7 south, m, k = 2. Top panels are scree plots used to select the best k. Bottom panels are: (left) including outliers, and (right) excluding outliers. Result: PC1 separates Chalifours from the other sites and Pe03 from Ic. PC2 seaprates Cha03 from 15. Removing outliers collapses Cha 03 and 15, and also collapses Pe03 completely into the other sites.

> print(ID[outliers])

[1] "866_78" "1137_78" "1745_76" "1910_79" "2364_79" "3183_76" "4431_19"

[8] "4885_78" "5302_79" "7221_52" "7583_78" "9415_78" "9664_69" "9942_79"

[15] "10957_77" "11235_78" "13218_79" "13833_79" "14431_79" "16571_28" "18719_79"

[22] "20292_25" "20378_78" "21844_79" "23629_79" "23745_79" "24957_79" "25144_79"

[29] "26667_79" "27021_71" "31135_79" "32440_79" "34289_47" "35231_79" "37447_48"

[36] "37605_43" "38039_35" "39974_49" "42257_74" "46581_77" "47695_78" "48658_18"

[43] "50030_36" "52351_78" "53498_56" "56508_40" "62534_37" "63490_78" "70726_79"

[50] "71059_77" "72944_78" "73353_79" "74122_76" "74788_54" "75276_78" "75947_43"

[57] "80019_79" "85905_79" "87113_79" "87783_79" "89820_78" "92110_79" "95210_79"

[64] "95709_79" "101352_79" "102737_78" "103420_43" "104310_77" "104441_34"

=69/8068 SNPs = 0.9% SNPs

PC2, 51 loci

> snp_pc

SNP PC

1 10 2

2 12 2

3 24 2

4 35 2

5 62 2

6 135 2

7 219 2

8 255 2

9 281 2

10 433 1

11 468 2

12 609 1

13 630 1

14 657 2

15 752 2

16 772 2

17 947 2

18 1010 2

19 1062 2

20 1238 1

21 1414 2

22 1552 1

23 1556 2

24 1671 2

25 1823 2

26 1835 2

27 1943 2

28 1959 2

29 2110 2

30 2138 1

31 2467 2

32 2580 2

33 2748 1

34 2845 2

35 3030 2

36 3045 1

37 3086 1

38 3228 2

39 3398 2

40 3737 2

41 3821 2

42 3888 1

43 3987 1

44 4125 2

45 4189 1

46 4344 1

47 4590 1

48 4650 1

49 5058 2

50 5092 2

51 5265 2

52 5301 2

53 5370 1

54 5422 2

55 5468 2

56 5526 2

57 5886 2

58 6406 2

59 6489 2

60 6548 2

61 6722 2

62 6916 2

63 7217 2

64 7270 2

65 7719 2

66 7831 2

67 7893 1

68 7979 2

69 7992 1


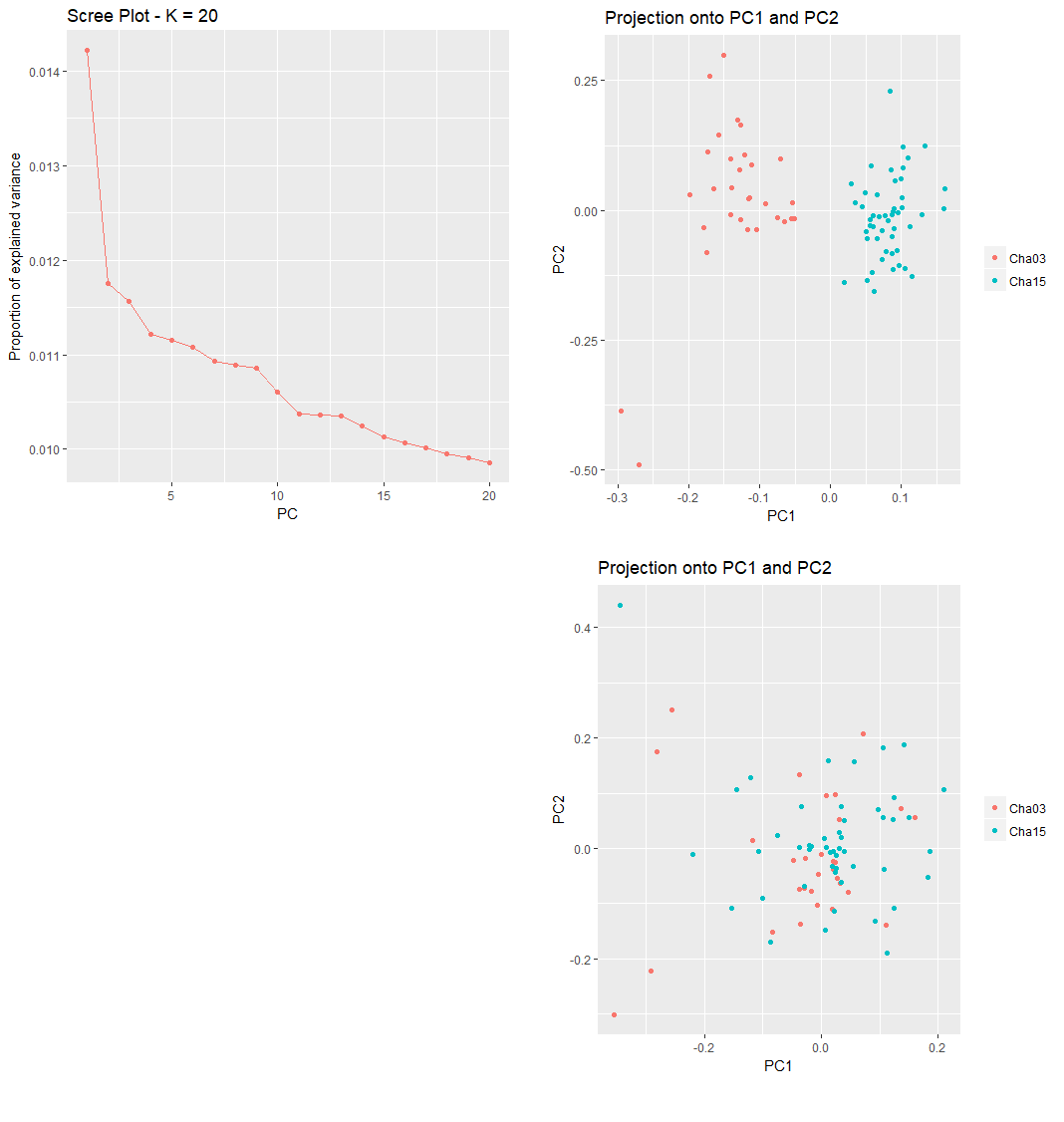


Figure S8 Chalifour, m and f combined. Upper left is scree plot showing that k=2 is best. Top right is including outliers, bottom right is excluding outliers. Outliers play a role in generating population structure within this river between years, shown by changes in population structure when outliers are excluded.

##list of outlier loci

[1] "866_78" "1137_78" "1721_78" "1910_79" "1972_79" "3995_79" "4975_78" "5410_79" "6284_10" "7083_79"

[11] "7205_77" "7393_79" "7583_78" "8039_8" "8206_79" "8525_79" "8979_6" "9415_78" "9985_79" "10151_77"

[21] "10154_65" "10696_78" "10903_77" "10957_77" "11235_78" "11521_76" "11606_77" "11815_79" "12116_77" "13160_47"

[31] "13218_79" "14431_79" "15115_77" "18075_79" "18117_78" "18719_79" "19386_76" "20090_61" "20218_69" "20708_79"

[41] "21651_73" "23670_78" "23745_79" "23907_79" "28075_79" "28547_78" "29693_79" "31717_34" "32583_79" "32700_77"

[51] "35231_79" "35262_76" "36788_78" "38592_75" "38867_58" "39743_77" "39974_49" "42735_20" "43313_77" "45325_76"

[61] "45642_79" "46439_76" "46825_12" "47058_78" "47354_76" "47695_78" "48159_76" "48329_79" "49162_60" "49921_75"

[71] "49954_34" "50404_26" "51477_59" "51976_28" "53073_79" "54508_79" "55665_78" "57110_77" "57652_79" "58632_29"

[81] "62869_78" "66231_29" "66290_62" "68634_75" "70511_54" "70882_78" "72447_18" "72620_77" "72944_78" "74712_73"

[91] "75225_79" "77093_45" "77273_78" "78044_40" "78090_11" "78754_11" "80019_79" "80680_79" "81351_78" "81730_77"

[101] "82672_77" "82817_79" "83973_79" "85905_79" "87141_78" "87342_79" "87508_12" "87783_79" "88453_78" "88537_30"

[111] "88666_78" "88895_78" "89820_78" "90763_79" "91449_77" "93522_79" "94770_78" "96420_52" "96940_76" "97531_77"

[121] "98454_43" "98768_69" "100129_79" "101049_10" "101352_79" "102737_78" "102740_43" "108226_76" "115082_57"

=129/8513 SNPs = 1.5%

##note that changing the number of PCs does change the number of outliers found.

#20 SNPs are associated with PC2

> snp_pc <- get.pc(x, outliers)

> snp_pc

SNP PC

1 18 1

2 21 1

3 36 1

4 48 1

5 53 1

6 208 1

7 277 1

8 305 1

9 384 2

10 454 1

11 467 1

12 491 1

13 512 1

14 555 1

15 566 1

16 590 1

17 624 2

18 665 1

19 723 1

20 741 1

21 742 1

22 794 1

23 814 1

24 822 1

25 842 1

26 865 1

27 875 1

28 895 1

29 915 1

30 1022 1

31 1027 1

32 1149 1

33 1216 1

34 1457 1

35 1463 1

36 1522 1

37 1579 1

38 1649 2

39 1659 2

40 1693 1

41 1780 1

42 1966 1

43 1973 1

44 1987 1

45 2371 1

46 2405 1

47 2503 1

48 2686 2

49 2769 1

50 2783 1

51 3027 1

52 3031 1

53 3159 1

54 3322 1

55 3346 1

56 3416 1

57 3433 2

58 3654 1

59 3707 1

60 3860 1

61 3882 1

62 3958 1

63 3980 2

64 4005 1

65 4029 1

66 4053 1

67 4088 1

68 4101 1

69 4164 1

70 4222 1

71 4225 2

72 4256 2

73 4328 2

74 4360 2

75 4421 1

76 4507 1

77 4551 1

78 4619 1

79 4651 1

80 4690 2

81 4870 1

82 5058 2

83 5064 1

84 5202 1

85 5319 1

86 5363 1

87 5505 1

88 5520 1

89 5557 1

90 5716 2

91 5759 1

92 5935 1

93 5954 1

94 6037 1

95 6042 1

96 6087 2

97 6202 1

98 6271 1

99 6345 1

100 6372 1

101 6474 1

102 6491 2

103 6592 1

104 6757 1

105 6843 1

106 6860 1

107 6872 2

108 6901 1

109 6971 1

110 6978 2

111 6993 1

112 7011 1

113 7087 1

114 7162 1

115 7225 1

116 7427 1

117 7559 1

118 7702 1

119 7748 1

120 7797 1

121 7870 1

122 7901 1

123 8025 1

124 8112 2

125 8132 1

126 8253 1

127 8254 1

128 8450 1

129 8486 2


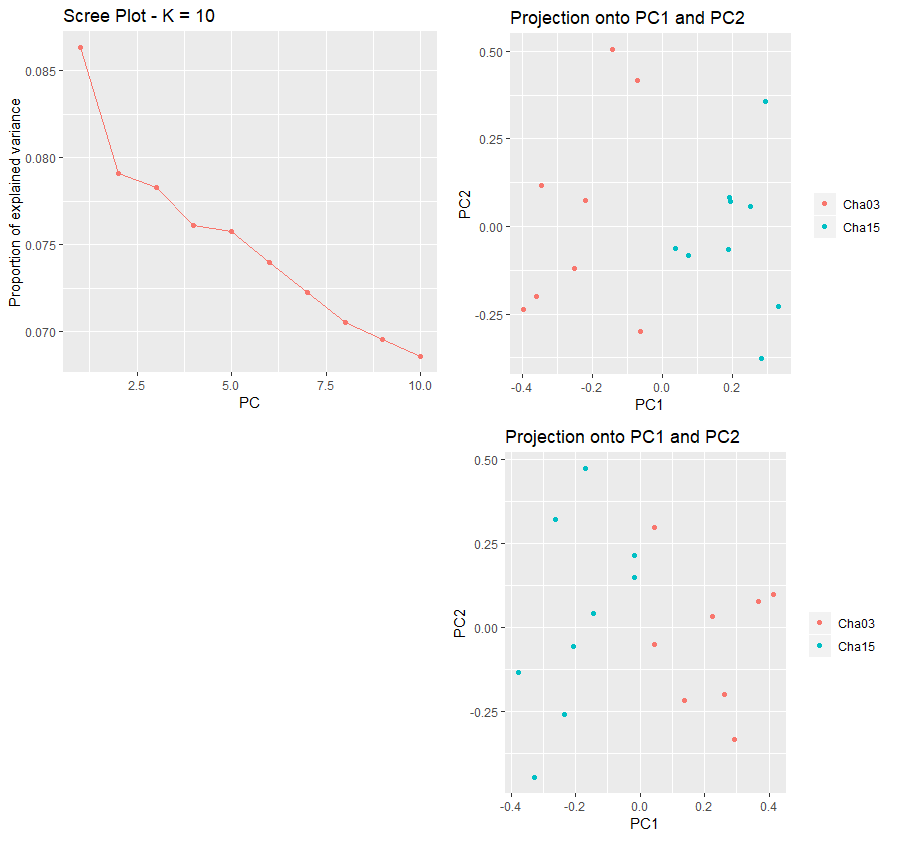


Figure S9 Chalifour f, k = 2. Top, with outliers, bottom, without outliers. There is sufficient structure to determine outliers. Based on the number of individuals it isn’t totally clear that the outliers are maintaining the structure present. Populations might overlap more when outliers are removed.

> print(ID[outliers])

[1] "3995_79" "5684_73" "7219_48" "8525_79" "12497_23" "15859_28" "20708_79" "21808_48" "23410_5" "23745_79"

[11] "28075_79" "31310_73" "39743_77" "46439_76" "46592_79" "51267_29" "72339_30" "76464_55" "76666_67" "79490_66"

[21] "81931_71" "88280_65" "90944_29" "96126_78" "103143_13"

SNP PC

1 94 1

2 141 1

3 201 2

4 248 1

5 401 2

6 525 1

7 704 1

8 740 1

9 801 1

10 815 1

11 1004 1

12 1138 2

13 1481 1

14 1721 1

15 1725 1

16 1890 1

17 2340 2

18 2498 2

19 2511 2

20 2620 2

21 2730 2

22 2979 2

23 3073 1

24 3319 1

25 3598 1

PC1, 16/25


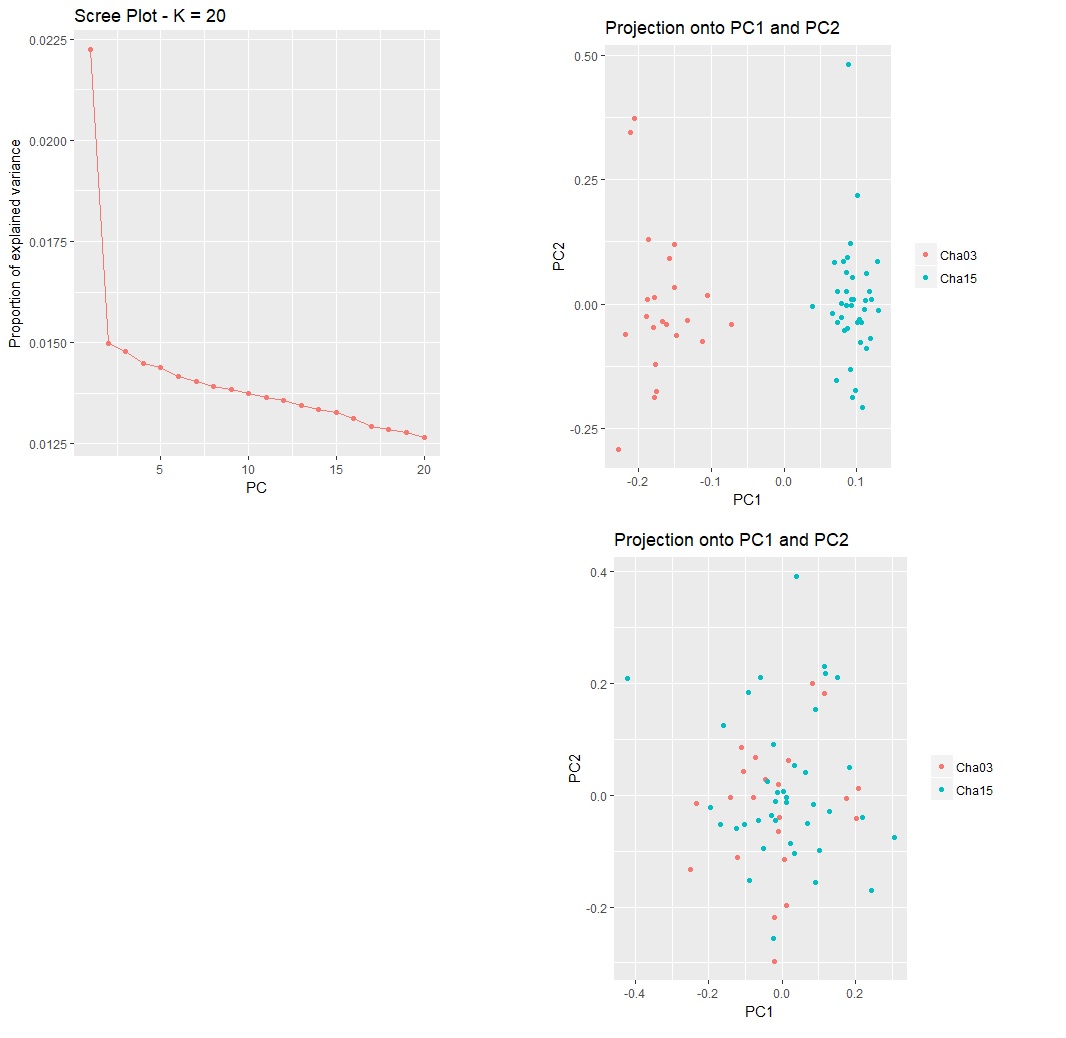


Figure S10 Chalifour m, k = 2. Top left panel is the scree plot showing best k. Top right is including outliers and lower right is excluding outliers. Result: PC1 separates the timepoints.

#outliers

[1] "866_78" "1137_78" "1721_78" "1745_79" "1910_79" "1972_76" "2776_77"

[8] "3049_79" "3084_15" "3995_78" "4066_36" "4431_19" "4885_78" "4975_78"

[15] "5155_43" "5302_79" "5410_79" "6056_10" "6527_51" "6583_79" "7083_79"

[22] "7205_77" "7393_79" "7664_78" "7828_36" "8039_8" "8206_78" "8521_79"

[29] "8525_79" "9283_28" "9290_77" "9415_78" "9629_79" "9942_79" "9985_79"

[36] "10154_65" "10157_79" "10696_78" "10903_77" "10916_78" "10957_77" "11235_78"

[43] "11433_78" "11521_76" "11761_37" "11815_79" "12116_77" "12894_79" "13192_59"

[50] "13218_79" "13263_13" "14431_76" "14895_79" "14996_79" "15115_77" "15863_78"

[57] "16462_79" "16510_18" "16851_79" "16860_79" "17897_78" "18075_78" "18117_78"

[64] "19036_78" "19262_77" "19386_76" "20154_78" "20357_79" "20708_79" "21844_79"

[71] "21874_78" "22119_76" "23670_78" "23745_79" "23771_79" "23907_79" "23960_64"

[78] "24304_78" "24957_79" "25144_79" "25199_77" "25396_43" "26211_77" "26578_78"

[85] "28026_42" "28547_78" "29069_79" "29208_61" "29284_77" "29693_79" "31135_79"

[92] "31509_74" "32228_78" "32421_46" "32583_79" "32700_77" "33919_79" "34382_79"

[99] "34542_79" "34842_79" "35231_79" "35267_78" "35878_58" "36142_29" "36714_75"

[106] "36788_78" "36958_77" "37237_77" "37307_55" "39371_78" "39866_79" "39983_22"

[113] "40111_28" "40299_7" "41112_79" "41251_79" "41774_78" "41835_79" "41986_78"

[120] "42673_76" "42823_69" "43077_77" "43313_77" "43351_78" "44182_45" "44786_79"

[127] "45325_76" "45557_77" "45594_57" "45642_79" "46825_12" "46963_72" "47058_78"

[134] "47354_76" "47468_79" "47695_78" "48329_79" "48513_31" "48737_77" "48833_75"

[141] "49162_60" "49364_39" "49548_30" "49921_75" "50987_25" "52351_78" "53073_79"

[148] "53362_77" "55882_46" "56093_48" "57110_77" "57652_79" "58649_77" "58827_77"

[155] "59518_78" "60277_76" "60974_79" "62211_78" "62222_79" "62869_78" "63988_78"

[162] "65789_33" "66676_29" "68634_75" "68770_77" "70693_79" "70699_56" "70882_78"

[169] "71655_77" "72007_79" "72079_78" "72447_18" "72620_77" "72944_78" "74712_73"

[176] "75225_79" "75276_78" "75295_75" "76810_78" "77032_78" "77088_77" "77093_45"

[183] "77332_78" "77709_78" "77786_79" "78044_40" "78552_47" "79015_78" "79047_76"

[190] "79613_79" "80019_79" "80664_78" "80680_79" "81320_50" "81351_78" "81405_79"

[197] "81596_78" "81671_78" "81730_77" "81745_23" "81922_79" "82349_79" "82672_77"

[204] "82679_76" "82892_78" "83863_67" "83973_79" "84062_75" "85260_79" "85504_79"

[211] "85977_63" "86067_79" "86409_78" "86842_79" "87113_79" "87141_78" "87276_75"

[218] "87342_79" "87476_79" "87758_78" "87783_79" "87972_79" "88453_78" "88749_71"

[225] "88815_79" "88895_78" "89780_78" "89820_78" "90422_77" "90459_77" "90763_78"

[232] "91449_77" "91852_78" "92230_78" "92855_35" "92867_79" "93140_19" "93437_61"

[239] "94159_79" "94442_79" "95224_79" "95709_79" "95720_79" "95961_15" "96076_79"

[246] "96533_78" "96940_76" "97365_77" "97657_76" "98454_43" "98768_69" "99817_38"

[253] "100129_79" "101352_79" "101890_19" "102377_79" "102505_79" "102737_78" "102857_77"

[260] "105475_78" "108226_76" "117457_61" "118233_49"

#that is a lot of outliers

=263/9294 SNPs = 2.8% of SNPs are outliers.

PC2, 30 loci

> snp_pc

SNP PC

1 17 1

2 20 1

3 36 1

4 37 1

5 50 1

6 55 1

7 119 1

8 154 1

9 159 2

10 228 1

11 237 2

12 270 1

13 305 1

14 310 1

15 324 2

16 332 1

17 341 1

18 410 1

19 457 1

20 462 1

21 502 1

22 517 1

23 541 1

24 571 1

25 586 2

26 607 1

27 618 1

28 643 1

29 644 1

30 705 1

31 706 1

32 721 1

33 742 1

34 781 1

35 784 1

36 803 1

37 804 1

38 857 1

39 877 1

40 878 1

41 886 1

42 909 1

43 934 1

44 939 1

45 962 2

46 973 1

47 993 1

48 1083 1

49 1117 2

50 1119 1

51 1127 2

52 1250 1

53 1307 1

54 1318 1

55 1327 1

56 1397 1

57 1458 1

58 1464 1

59 1499 1

60 1500 1

61 1583 1

62 1603 1

63 1611 1

64 1707 1

65 1726 1

66 1739 1

67 1825 1

68 1840 1

69 1869 1

70 1975 1

71 1980 1

72 2010 1

73 2162 1

74 2173 1

75 2175 1

76 2190 1

77 2196 1

78 2231 1

79 2297 1

80 2316 1

81 2321 1

82 2340 1

83 2427 1

84 2478 1

85 2598 1

86 2640 1

87 2694 1

88 2706 2

89 2714 1

90 2749 1

91 2894 1

92 2925 1

93 2997 1

94 3018 2

95 3034 1

96 3048 1

97 3170 1

98 3215 1

99 3234 1

100 3268 1

101 3312 1

102 3317 1

103 3376 1

104 3406 2

105 3450 1

106 3458 1

107 3475 1

108 3506 1

109 3516 2

110 3708 1

111 3753 1

112 3762 1

113 3773 2

114 3789 1

115 3855 1

116 3869 1

117 3910 1

118 3921 1

119 3930 1

120 3995 2

121 4006 2

122 4030 1

123 4055 1

124 4061 1

125 4129 1

126 4179 1

127 4227 1

128 4247 1

129 4249 1

130 4252 1

131 4353 1

132 4370 2

133 4379 1

134 4405 1

135 4413 1

136 4428 1

137 4477 1

138 4493 2

139 4511 1

140 4521 1

141 4544 1

142 4554 2

143 4571 2

144 4602 1

145 4679 2

146 4779 1

147 4826 1

148 4842 1

149 4989 1

150 5000 2

151 5051 1

152 5083 1

153 5129 1

154 5139 1

155 5156 1

156 5202 1

157 5234 1

158 5292 1

159 5294 1

160 5329 1

161 5394 1

162 5511 2

163 5571 1

164 5696 1

165 5704 1

166 5841 1

167 5842 1

168 5865 1

169 5940 1

170 5977 1

171 5987 1

172 6019 1

173 6036 1

174 6072 1

175 6240 2

176 6290 1

177 6299 1

178 6302 1

179 6453 1

180 6478 1

181 6485 1

182 6486 1

183 6512 1

184 6553 1

185 6560 1

186 6593 1

187 6631 2

188 6679 1

189 6681 1

190 6741 1

191 6777 1

192 6849 1

193 6851 1

194 6927 2

195 6930 1

196 6932 1

197 6950 1

198 6955 1

199 6959 1

200 6961 2

201 6987 1

202 7033 1

203 7070 1

204 7072 1

205 7098 1

206 7185 1

207 7198 1

208 7202 1

209 7302 1

210 7327 1

211 7378 1

212 7382 1

213 7412 1

214 7442 1

215 7464 1

216 7467 1

217 7477 1

218 7485 1

219 7497 1

220 7526 1

221 7528 1

222 7546 1

223 7601 1

224 7628 2

225 7636 1

226 7644 1

227 7725 1

228 7729 1

229 7792 1

230 7796 1

231 7818 1

232 7885 1

233 7922 1

234 7966 1

235 8025 1

236 8028 1

237 8061 2

238 8091 1

239 8168 1

240 8194 1

241 8271 1

242 8324 1

243 8326 1

244 8340 2

245 8352 2

246 8396 1

247 8431 1

248 8478 1

249 8499 1

250 8568 1

251 8600 1

252 8697 1

253 8721 1

254 8840 1

255 8879 2

256 8929 1

257 8943 1

258 8965 1

259 8978 1

260 9169 1

261 9178 1

262 9260 1

263 9267 1


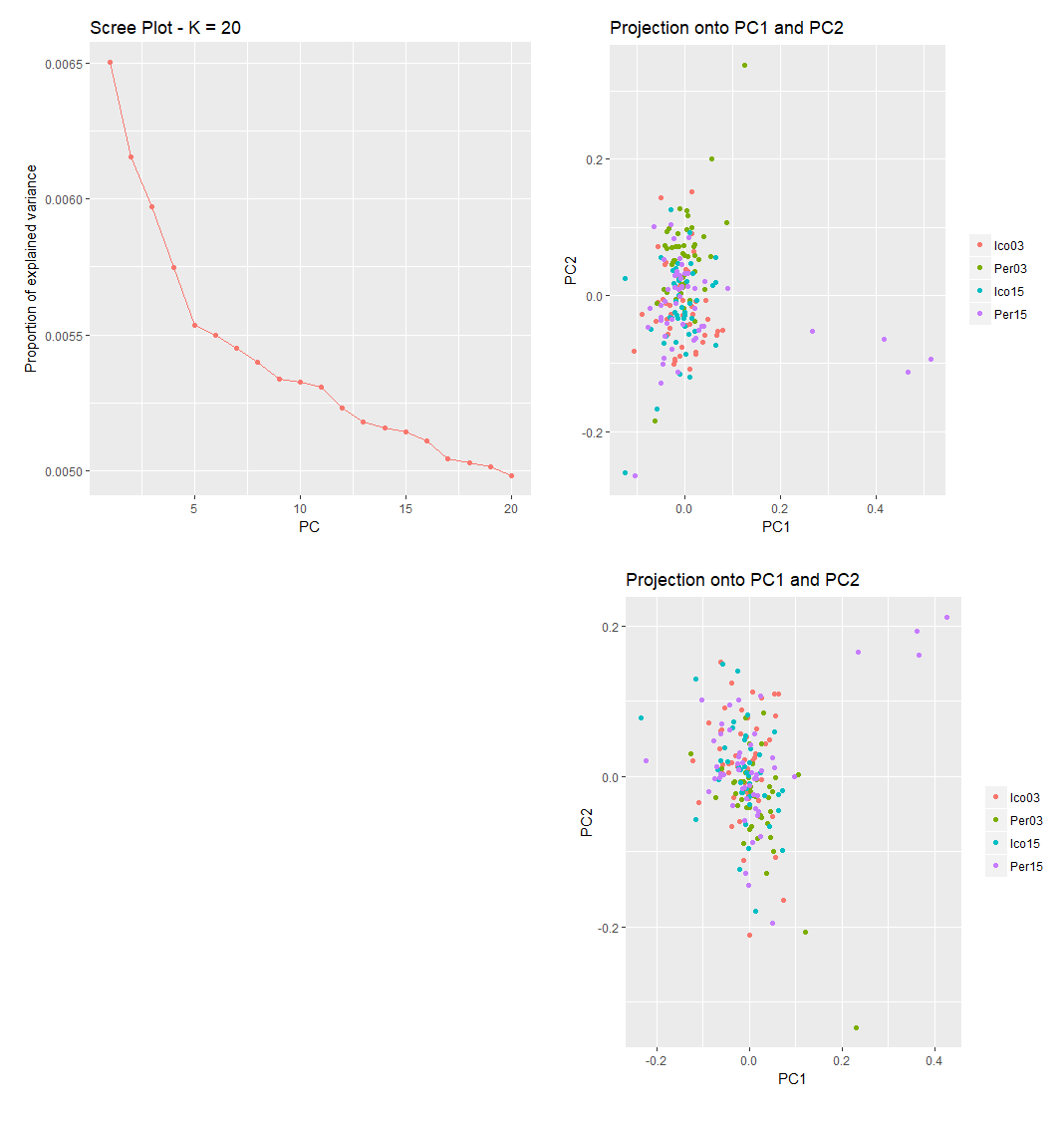


Figure S11 Ic-Pe, m and f combined. Top left is the scree plot showing that k = 5 looks best. However, contribution of PC3 to population structure was tested, and it does not change the conclusion. Thus, k=2 was chosen. Top right is with outlier loci included, and bottom right is with outlier loci excluded. Conclusion: although structure is relatively weak within Ic-Pe between years, there is structure, and outlier loci contribute to this structure. Pe03 separates out on PC2. In SNP list associated with PCs below, you can see that 8 SNPs are associated with this PC.

> print(ID[outliers])

[1] "3718_64" "7174_45" "10957_77" "16314_49" "18923_13" "19294_69" "20378_78" "23360_77"

[9] "31135_79" "31873_28" "32359_23" "33513_45" "35165_18" "41903_57" "43027_6" "44920_15"

[17] "46714_51" "50605_54" "54214_10" "66470_39" "72908_11" "73469_62" "73918_69" "77907_75"

[25] "79366_26" "80339_30" "86537_49" "86863_71" "87113_79" "93777_10" "96469_21" "96847_31"

[33] "99581_77"

=33/6802 = 0.49% of loci are outliers. Note that there are 51 outliers when k=5 is used. When k=5 is used, there are some SNPs that assign to Pcs 3, 4 and 5.

SNP PC

1 141 2

2 352 1

3 613 2

4 1000 1

5 1179 1

6 1206 1

7 1294 2

8 1502 2

9 2089 2

10 2142 1

11 2177 1

12 2277 1

13 2405 1

14 2851 1

15 2929 2

16 3048 1

17 3158 1

18 3403 1

19 3561 1

20 4016 1

21 4400 1

22 4441 1

23 4471 1

24 4771 1

25 4877 1

26 4959 1

27 5409 1

28 5424 1

29 5437 2

30 5970 1

31 6180 1

32 6203 1

33 6399 2


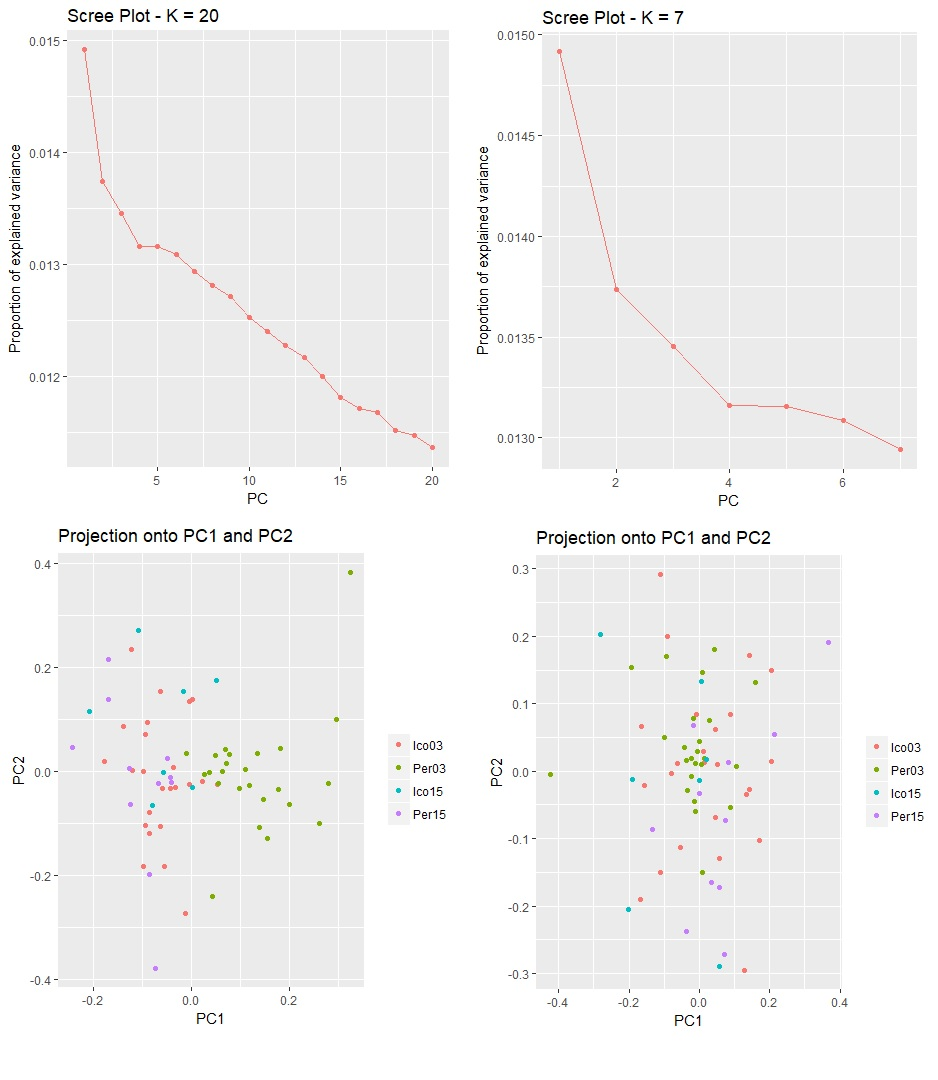


Figure S12 Ic-Pe f, k=2. Top panels are scree plots used to determine best k. Bottom plots are (right) with outlier loci included, and (left) excluding outlier loci. Result: PC1 separates Pe03 from the other populations.

> print(ID[outliers])

[1] "639_75" "3193_79" "4975_78" "7583_78" "8169_79" "8206_79" "10957_77"

[8] "13323_72" "14431_76" "16324_79" "18171_79" "18297_78" "19848_54" "19893_60"

[15] "23360_77" "23519_75" "24836_77" "24957_79" "25199_77" "27499_78" "28075_78"

[22] "28601_43" "28887_76" "29693_78" "31509_74" "32239_7" "32700_77" "32851_77"

[29] "34803_77" "34842_79" "35262_76" "35267_78" "41258_78" "41419_78" "42767_66"

[36] "45557_77" "46292_78" "46573_78" "47695_78" "48757_78" "49415_76" "50171_79"

[43] "55665_78" "61999_58" "62150_77" "64088_78" "70726_79" "71278_78" "72944_79"

[50] "73353_79" "73469_62" "75276_78" "75331_77" "76823_79" "77966_78" "83973_79"

[57] "84062_75" "87476_79" "88895_78" "89347_55" "89405_57" "94671_78" "94835_32"

[64] "96076_79" "96289_77" "97365_77" "98249_5" "99081_19" "99581_77" "99999_73"

[71] "102675_77" "104310_77" "104326_23"

##whoa, that is a lot of outliers. 73/6442 SNPs = 1.1% of SNPs

##note that this is the same result as we saw with both males and females included in the dataset

> snp_pc <- get.pc(x, outliers)

> snp_pc

SNP PC

1 11 1

2 120 1

3 214 1

4 363 1

5 396 1

6 398 1

7 560 1

8 802 1

9 918 1

10 1044 1

11 1396 1

12 1411 1

13 1435 2

14 1510 1

15 1520 1

16 1537 1

17 1660 2

18 1708 1

19 1741 1

20 1775 1

21 1800 1

22 1854 1

23 1997 1

24 2050 2

25 2086 1

26 2104 1

27 2247 1

28 2250 1

29 2282 1

30 2283 1

31 2639 2

32 2660 1

33 2669 1

34 2670 1

35 2914 1

36 2963 1

37 2972 1

38 3046 1

39 3106 1

40 3148 1

41 3193 1

42 3405 1

43 3612 1

44 3685 1

45 3789 2

46 3982 1

47 4032 1

48 4150 1

49 4180 1

50 4270 1

51 4311 1

52 4317 1

53 4383 2

54 4412 1

55 4512 1

56 4950 1

57 4953 1

58 5170 1

59 5280 1

60 5337 1

61 5683 1

62 5717 1

63 5768 2

64 5824 1

65 5837 1

66 5918 1

67 6025 2

68 6031 1

69 6057 1

70 6091 2

71 6256 1

72 6392 1

73 6394 2


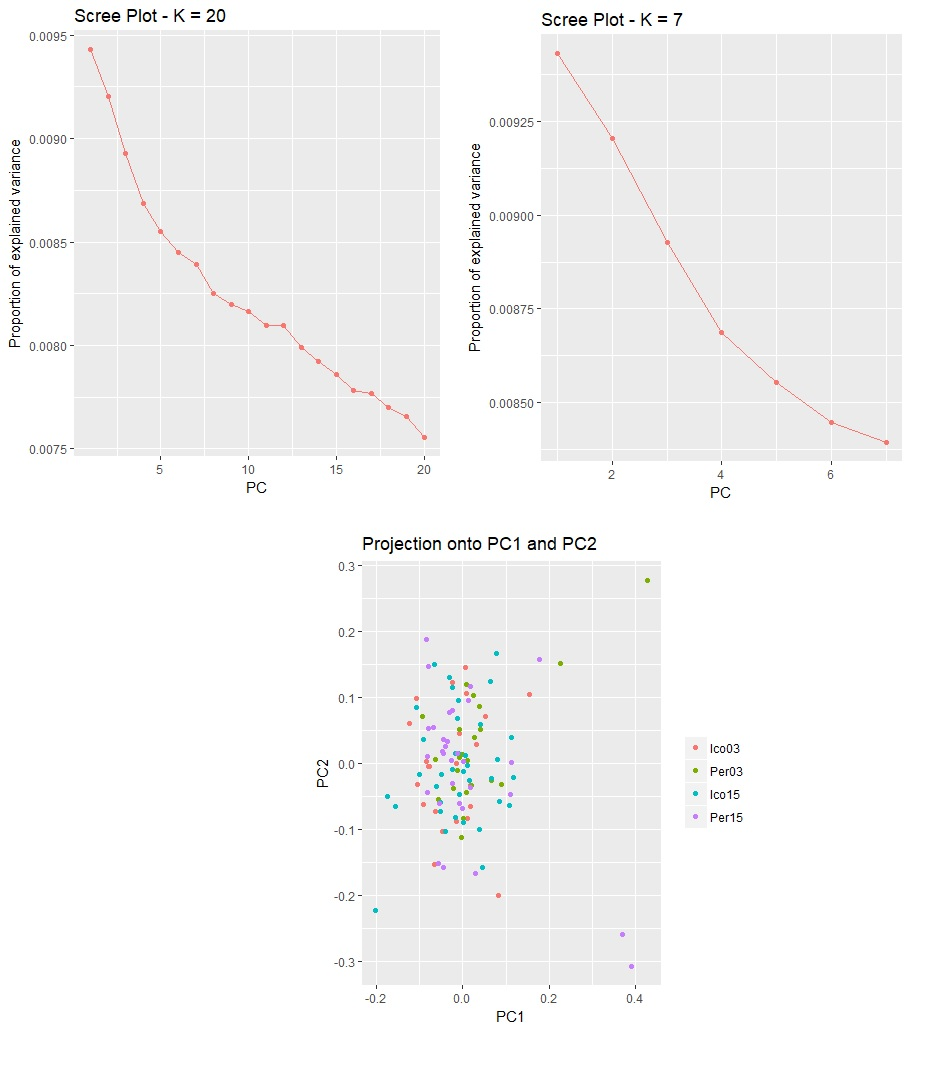


Figure S13 Ic-Pe m, k = 4. Top panels are scree plots showing that there is insufficient structure to find a best k. Bottom panel is including outliers. Both k = 4 and k = 6 were tested with the same result.


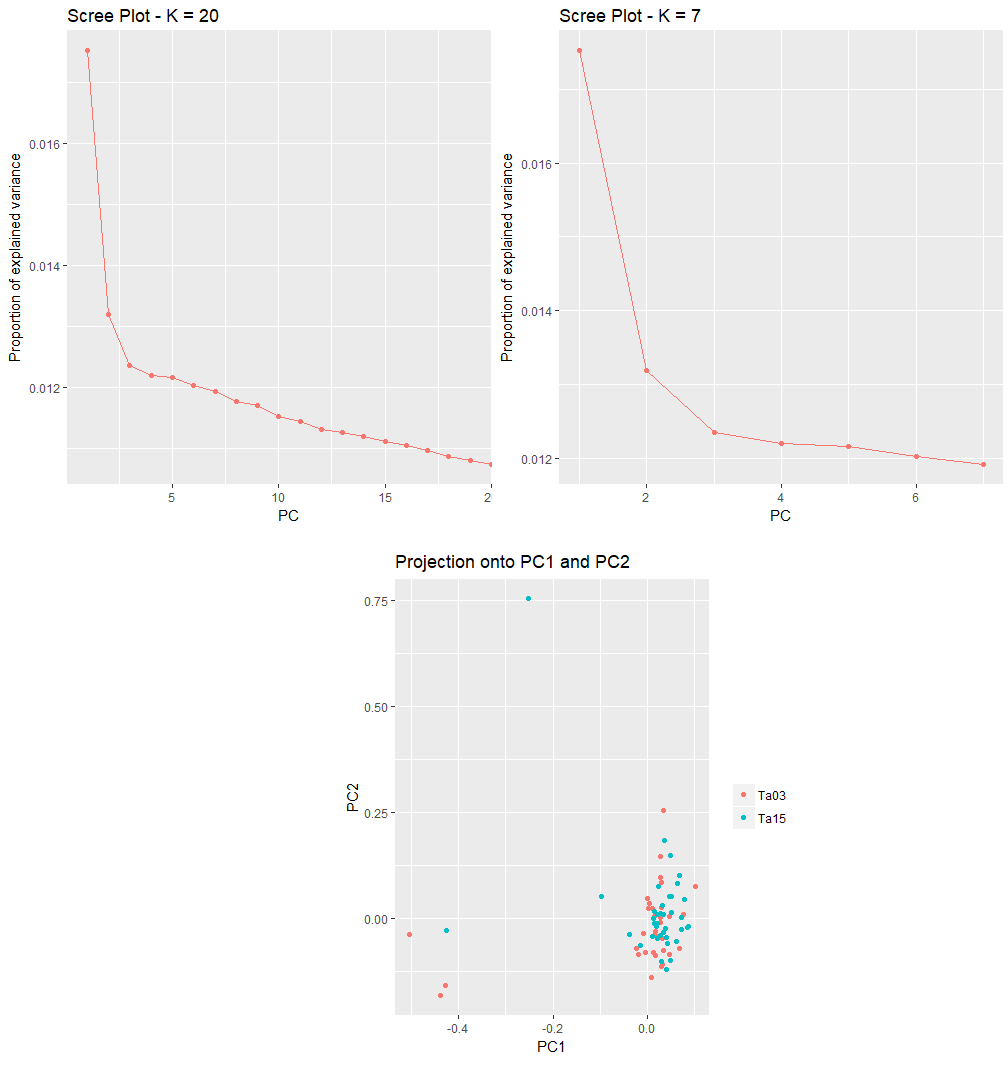


Figure S14 Takwa, m and f combined k=2, The top panel is scree plots showing the best k. Bottom panel is with outliers included, and shows that there is no structure between years. The structure seen in the scree plot is likely due to those outlier individuals.


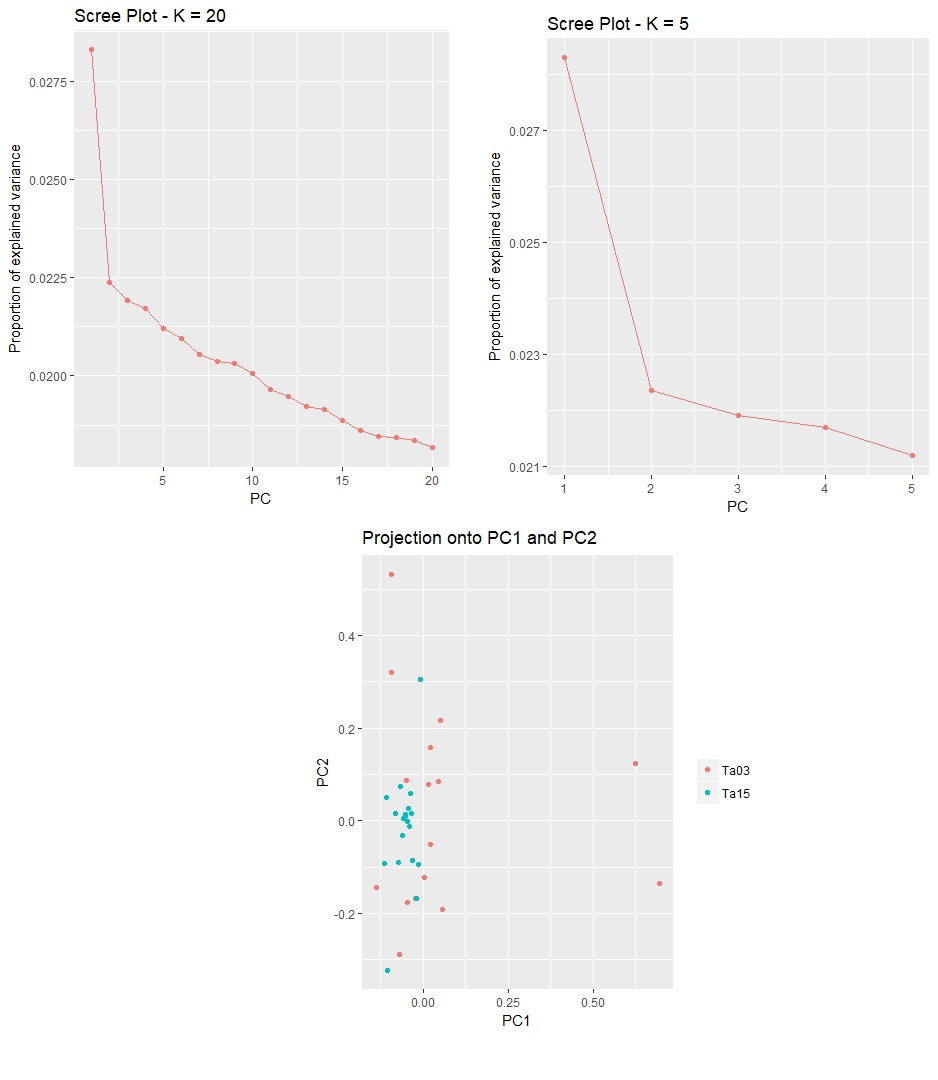


Figure S15 Takwa River, f, k=2. Top panels are scree plots used to find the best k. Bottom panel is including outliers. Result: There is no structure between years within Takwa River. Outliers cannot be assessed.


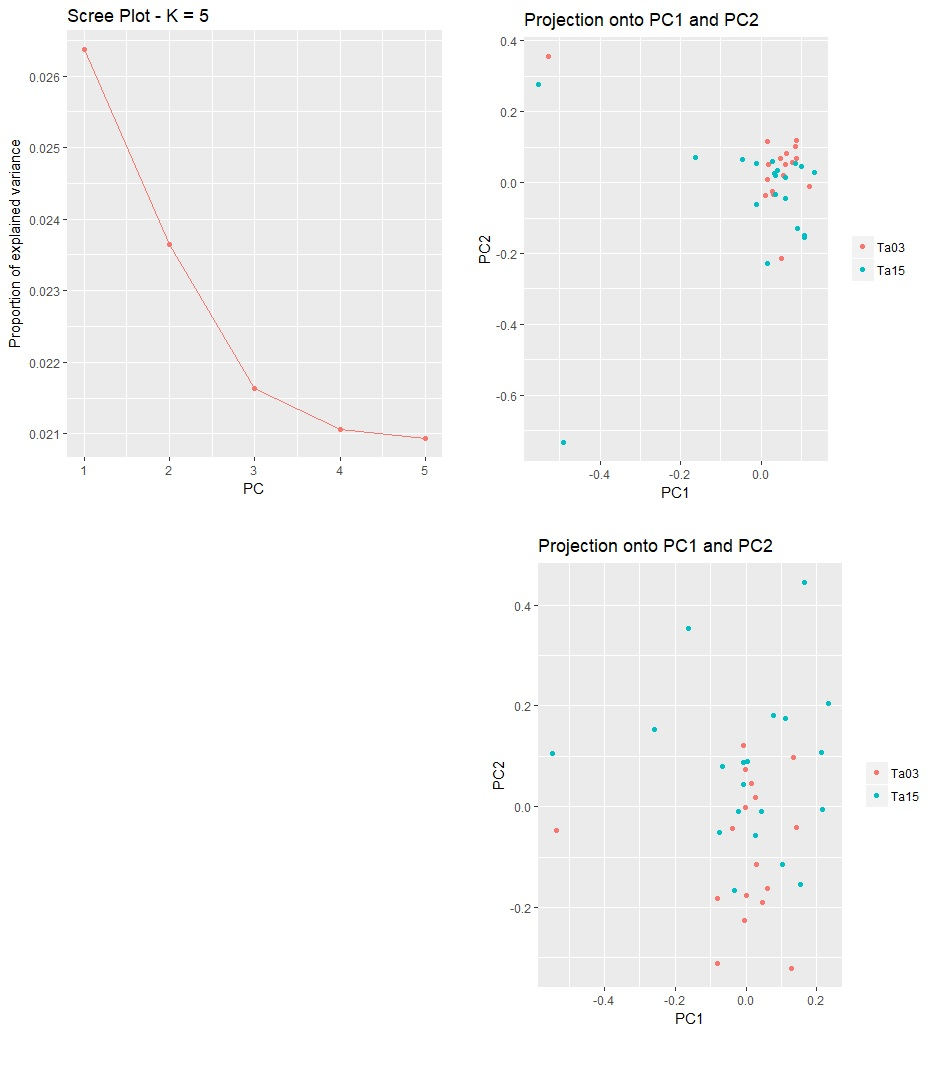


Figure S16 Takwa m, k=3. Top left panel showing scree plot used to find best k. Top right panel is including outliers. Bottom right panel is excluding outliers. Result: there is no population structure between years, and thus no outliers can be found.

Outlier loci in common

Comparing A (all_all) and B, (ALL_F): The sets have 8 SNPs in common:[1] "2764_47" "16571_28" "34289_47" "36864_48" "45711_42" "89842_9" "93560_78" "103420_43"

Comparing A (all_all) and C, (all, M): The sets have 59 SNPs in common: [1] "2764_47" "5777_26" "7221_52" "9664_69" "10060_79" "10881_53" "11507_79" "12647_55"

[9] "15362_57" "15426_49" "16571_28" "23892_66" "25728_47" "26281_46" "27021_71" "28192_35"

[17] "29788_76" "31886_71" "35235_60" "36864_48" "37605_43" "38039_35" "39685_62" "41527_40"

[25] "41635_58" "45711_42" "46331_65" "48658_18" "49504_49" "50030_36" "50404_26" "51264_20"

[33] "53498_56" "62465_26" "65438_60" "65770_56" "69567_54" "71554_26" "76517_75" "81466_14"

[41] "83593_61" "84174_76" "85630_70" "88020_72" "88250_71" "89842_9" "91022_14" "91315_70"

[49] "91507_23" "93560_78" "93677_42" "97401_34" "97840_17" "99888_60" "101083_6" "102928_19"

[57] "103420_43" "104231_8" "104441_34"

Comparing A (all_all) and D (sou, all): The sets have 9 SNPs in common:[1] "16571_28" "27021_71" "34289_47" "37605_43" "48658_18" "53498_56" "74122_76" "103420_43"

[9] "104441_34"

Comparing A (all_all) and E, (SOU_F): The sets have 2 SNPs in common:[1] "16571_28" "34289_47"

Comparing A (all_all) and F: The sets have 13 SNPs in common: [1] "7221_52" "9664_69" "16571_28" "27021_71" "34289_47" "37605_43" "38039_35" "48658_18"

[9] "50030_36" "53498_56" "74122_76" "103420_43" "104441_34"

Comparing A (all_all) and G, (IC-PE, ALL): The sets have 0 SNPs in common:character(0)

Comparing A (all_all) and H, (IC-PE, F): The sets have 0 SNPs in common:character(0)

Comparing A (all_all) and I, (CH_ALL): The sets have 1 SNPs in common:[1] "50404_26"

Comparing A (all_all) and J, (CH_F): The sets have 0 SNPs in common:character(0)

Comparing A (all_all) and K, (CH_M): The sets have 0 SNPs in common:character(0)

Comparing B, (ALL_F) and C , (all, M): The sets have 7 SNPs in common:[1] "2764_47" "16571_28" "36864_48" "45711_42" "89842_9" "93560_78" "103420_43"

Comparing B, (ALL_F) and D (sou, all): The sets have 3 SNPs in common:[1] "16571_28" "34289_47" "103420_43"

Comparing B, (ALL_F) and E (SOU_F), (SOU_F): The sets have 3 SNPs in common:[1] "16571_28" "27138_61" "34289_47"

Comparing B, (ALL_F) and F: The sets have 3 SNPs in common:[1] "16571_28" "34289_47" "103420_43"

Comparing B, (ALL_F) and G, (IC-PE, ALL): The sets have 0 SNPs in common:character(0)

Comparing B, (ALL_F) and H, (IC-PE, F): The sets have 0 SNPs in common:character(0)

Comparing B, (ALL_F) and I, (CH_ALL): The sets have 0 SNPs in common:character(0)

Comparing B, (ALL_F) and J, (CH_F): The sets have 0 SNPs in common:character(0)

Comparing B, (ALL_F) and K, (CH_M): The sets have 0 SNPs in common:character(0)

Comparing C, (all, M) and D: The sets have 15 SNPs in common: [1] "9415_78" "9942_79" "16571_28" "27021_71" "31135_79" "34803_77" "34842_79" "37605_43"

[9] "48658_18" "53498_56" "62534_37" "72944_78" "87113_79" "103420_43" "104441_34"

Comparing C, (all, M) and E, (SOU_F): The sets have 3 SNPs in common:[1] "16571_28" "34803_77" "34842_79"

Comparing C, (all, M) and F (SOU_M): The sets have 20 SNPs in common: [1] "7221_52" "9415_78" "9664_69" "9942_79" "16571_28" "21844_79" "26667_79" "27021_71"

[9] "31135_79" "37605_43" "38039_35" "48658_18" "50030_36" "53498_56" "62534_37" "72944_78"

[17] "87113_79" "87783_79" "103420_43" "104441_34"

Comparing C, (all, M) and G, (IC-PE, ALL): The sets have 2 SNPs in common:[1] "31135_79" "87113_79"

Comparing C, (all, M) and H, (IC-PE, F): The sets have 4 SNPs in common:[1] "14431_76" "34803_77" "34842_79" "45557_77"

Comparing C, (all, M) and I, (CH_ALL): The sets have 10 SNPs in common: [1] "1721_78" "7393_79" "9415_78" "10696_78" "50404_26" "72447_18" "72944_78" "87783_79"

[9] "96940_76" "101049_10"

Comparing C, (all, M) and J, (CH_F): The sets have 0 SNPs in common:character(0)

Comparing C, (all, M) and K, (CH_M): The sets have 37 SNPs in common: [1] "1721_78" "7393_79" "9415_78" "9629_79" "9942_79" "10157_79" "10696_78" "14431_76" "19262_77"

[10] "21844_79" "23960_64" "31135_79" "32228_78" "34382_79" "34842_79" "41986_78" "43077_77" "43351_78"

[19] "44786_79" "45557_77" "59518_78" "63988_78" "71655_77" "72447_18" "72944_78" "79047_76" "80664_78"

[28] "82349_79" "82679_76" "86067_79" "87113_79" "87783_79" "87972_79" "90459_77" "91852_78" "94159_79"

[37] "96940_76"

Comparing D, (SOU_ALL) and E, (SOU_F): The sets have 23 SNPs in common: [1] "3193_79" "4975_78" "7583_78" "10957_77" "16571_28" "23360_77" "24957_79" "28075_78"

[9] "29693_78" "34289_47" "34803_77" "34842_79" "35262_76" "47695_78" "70726_79" "74568_79"

[17] "75276_78" "83973_79" "88895_78" "92110_78" "96076_79" "99202_76" "104310_77"

Comparing D, (SOU_ALL) and F (SOU_M): The sets have 27 SNPs in common: [1] "866_78" "5302_79" "7583_78" "9415_78" "9942_79" "10957_77" "11235_78" "16571_28"

[9] "24957_79" "27021_71" "31135_79" "34289_47" "37605_43" "47695_78" "48658_18" "53498_56"

[17] "56508_40" "62534_37" "70726_79" "72944_78" "74122_76" "75276_78" "87113_79" "89820_78"

[25] "103420_43" "104310_77" "104441_34"

Comparing D, (SOU_ALL) and G, (IC-PE, ALL): The sets have 4 SNPs in common:[1] "10957_77" "23360_77" "31135_79" "87113_79"

Comparing D, (SOU_ALL) and H, (IC-PE, F): The sets have 18 SNPs in common: [1] "3193_79" "4975_78" "7583_78" "10957_77" "23360_77" "24957_79" "28075_78" "29693_78"

[9] "34803_77" "34842_79" "35262_76" "47695_78" "70726_79" "75276_78" "83973_79" "88895_78"

[17] "96076_79" "104310_77"

Comparing D, (SOU_ALL) and I, (CH_ALL): The sets have 12 SNPs in common: [1] "866_78" "4975_78" "7583_78" "9415_78" "10957_77" "11235_78" "35262_76" "47695_78" "72944_78"

[10] "83973_79" "88895_78" "89820_78"

Comparing D, (SOU_ALL) and J, (CH_F): The sets have 0 SNPs in common:character(0)

Comparing D, (SOU_ALL) and K, (CH_M): The sets have 20 SNPs in common: [1] "866_78" "4975_78" "5302_79" "8206_78" "9415_78" "9942_79" "10957_77" "11235_78" "24957_79"

[10] "29208_61" "31135_79" "34842_79" "47695_78" "72944_78" "75276_78" "83973_79" "87113_79" "88895_78"

[19] "89820_78" "96076_79"

Comparing E, (SOU_F) and F (SOU_M): The sets have 9 SNPs in common:[1] "7583_78" "10957_77" "16571_28" "24957_79" "34289_47" "47695_78" "70726_79" "75276_78"

[9] "104310_77"

Comparing E, (SOU_F) and G, (IC-PE, ALL): The sets have 2 SNPs in common:[1] "10957_77" "23360_77"

Comparing E, (SOU_F) and H, (IC-PE, F): The sets have 31 SNPs in common: [1] "3193_79" "4975_78" "7583_78" "8206_79" "10957_77" "18297_78" "23360_77" "23519_75"

[9] "24957_79" "28075_78" "29693_78" "31509_74" "32700_77" "34803_77" "34842_79" "35262_76"

[17] "41258_78" "46573_78" "47695_78" "48757_78" "50171_79" "55665_78" "62150_77" "70726_79"

[25] "71278_78" "75276_78" "83973_79" "88895_78" "96076_79" "102675_77" "104310_77"

Comparing E, (SOU_F) and I, (CH_ALL): The sets have 14 SNPs in common: [1] "4975_78" "7583_78" "8206_79" "10957_77" "11815_79" "32700_77" "35262_76" "46439_76" "47695_78"

[10] "55665_78" "77273_78" "80680_79" "83973_79" "88895_78"

Comparing E, (SOU_F) and J, (CH_F): The sets have 2 SNPs in common:[1] "46439_76" "46592_79"

Comparing E, (SOU_F) and K, (CH_M): The sets have 13 SNPs in common: [1] "4975_78" "10957_77" "11815_79" "24957_79" "31509_74" "32700_77" "34842_79" "47695_78" "75276_78"

[10] "80680_79" "83973_79" "88895_78" "96076_79"

Comparing F, (SOU_M) and G, (IC-PE, ALL): The sets have 4 SNPs in common:[1] "10957_77" "20378_78" "31135_79" "87113_79"

Comparing F, (SOU_M) and H, (IC-PE, F): The sets have 8 SNPs in common:[1] "7583_78" "10957_77" "24957_79" "47695_78" "70726_79" "73353_79" "75276_78" "104310_77"

Comparing F, (SOU_M) and I, (CH_ALL), (CH_ALL): The sets have 21 SNPs in common: [1] "866_78" "1137_78" "1910_79" "7583_78" "9415_78" "10957_77" "11235_78" "13218_79"

[9] "14431_79" "18719_79" "23745_79" "35231_79" "39974_49" "47695_78" "72944_78" "80019_79"

[17] "85905_79" "87783_79" "89820_78" "101352_79" "102737_78"

Comparing F, (SOU_M) and J, (CH_F): The sets have 1 SNPs in common:[1] "23745_79"

Comparing F, (SOU_M) and K, (CH_M): The sets have 28 SNPs in common: [1] "866_78" "1137_78" "1910_79" "4431_19" "4885_78" "5302_79" "9415_78" "9942_79"

[9] "10957_77" "11235_78" "13218_79" "21844_79" "23745_79" "24957_79" "25144_79" "31135_79"

[17] "35231_79" "47695_78" "52351_78" "72944_78" "75276_78" "80019_79" "87113_79" "87783_79"

[25] "89820_78" "95709_79" "101352_79" "102737_78"

####the most interesting follow from here

Comparing G, (IC-PE, ALL) and H, (IC-PE, F): The sets have 4 SNPs in common:[1] "10957_77" "23360_77" "73469_62" "99581_77"

Comparing G, (IC-PE, ALL) and I, (CH_ALL): The sets have 1 SNPs in common:[1] "10957_77"

Comparing G, (IC-PE, ALL) and J, (CH_F): The sets have 0 SNPs in common:character(0)

Comparing G, (IC-PE, ALL) and K, (CH_M): The sets have 3 SNPs in common:[1] "10957_77" "31135_79" "87113_79"

Comparing H, (IC-PE, F) and I, (CH_ALL): The sets have 10 SNPs in common: [1] "4975_78" "7583_78" "8206_79" "10957_77" "32700_77" "35262_76" "47695_78" "55665_78" "83973_79"

[10] "88895_78"

Comparing H, (IC-PE, F) and J, (CH_F): The sets have 0 SNPs in common:character(0)

Comparing H, (IC-PE, F) and K, (CH_M): The sets have 18 SNPs in common: [1] "4975_78" "10957_77" "14431_76" "24957_79" "25199_77" "31509_74" "32700_77" "34842_79" "35267_78"

[10] "45557_77" "47695_78" "75276_78" "83973_79" "84062_75" "87476_79" "88895_78" "96076_79" "97365_77"

Comparing I, (CH_ALL) and J, (CH_F): The sets have 7 SNPs in common:[1] "3995_79" "8525_79" "20708_79" "23745_79" "28075_79" "39743_77" "46439_76"

Comparing I, (CH_ALL) and K, (CH_M): The sets have 78 SNPs in common: [1] "866_78" "1137_78" "1721_78" "1910_79" "4975_78" "5410_79" "7083_79" "7205_77"

[9] "7393_79" "8039_8" "8525_79" "9415_78" "9985_79" "10154_65" "10696_78" "10903_77"

[17] "10957_77" "11235_78" "11521_76" "11815_79" "12116_77" "13218_79" "15115_77" "18117_78"

[25] "19386_76" "20708_79" "23670_78" "23745_79" "23907_79" "28547_78" "29693_79" "32583_79"

[33] "32700_77" "35231_79" "36788_78" "43313_77" "45325_76" "45642_79" "46825_12" "47058_78"

[41] "47354_76" "47695_78" "48329_79" "49162_60" "49921_75" "53073_79" "57110_77" "57652_79"

[49] "62869_78" "68634_75" "70882_78" "72447_18" "72620_77" "72944_78" "74712_73" "75225_79"

[57] "77093_45" "78044_40" "80019_79" "80680_79" "81351_78" "81730_77" "82672_77" "83973_79"

[65] "87141_78" "87342_79" "87783_79" "88453_78" "88895_78" "89820_78" "91449_77" "96940_76"

[73] "98454_43" "98768_69" "100129_79" "101352_79" "102737_78" "108226_76"

Comparing J, (CH_F) and K, (CH_M): The sets have 3 SNPs in common:[1] "8525_79" "20708_79" "23745_79"
